# Association of Variants in the *SPTLC1* Gene With Juvenile Amyotrophic Lateral Sclerosis

**DOI:** 10.1001/jamaneurol.2021.2598

**Published:** 2021-08-30

**Authors:** Janel O. Johnson, Ruth Chia, Danny E. Miller, Rachel Li, Ravindran Kumaran, Yevgeniya Abramzon, Nada Alahmady, Alan E. Renton, Simon D. Topp, J. Raphael Gibbs, Mark R. Cookson, Marya S. Sabir, Clifton L. Dalgard, Claire Troakes, Ashley R. Jones, Aleksey Shatunov, Alfredo Iacoangeli, Ahmad Al Khleifat, Nicola Ticozzi, Vincenzo Silani, Cinzia Gellera, Ian P. Blair, Carol Dobson-Stone, John B. Kwok, Emily S. Bonkowski, Robin Palvadeau, Pentti J. Tienari, Karen E. Morrison, Pamela J. Shaw, Ammar Al-Chalabi, Robert H. Brown, Andrea Calvo, Gabriele Mora, Hind Al-Saif, Marc Gotkine, Fawn Leigh, Irene J. Chang, Seth J. Perlman, Ian Glass, Anna I. Scott, Christopher E. Shaw, A. Nazli Basak, John E. Landers, Adriano Chiò, Thomas O. Crawford, Bradley N. Smith, Bryan J. Traynor, and the FALS Sequencing Consortium; American Genome Center; International ALS Genomics Consortium; and ITALSGEN Consortium, Bradley N. Smith, Nicola Ticozzi, Claudia Fallini, Athina Soragia Gkazi, Simon D. Topp, Emma L. Scotter, Kevin P. Kenna, Pamela Keagle, Cinzia Tiloca, Caroline Vance, Claire Troakes, Claudia Colombrita, Andrew King, Viviana Pensato, Barbara Castellotti, Frank Baas, Anneloor L. M. A. ten Asbroek, Diane McKenna-Yasek, Russell L. McLaughlin, Meraida Polak, Seneshaw Asress, Jesús Esteban-Pérez, Zorica Stevic, Sandra D’Alfonso, Letizia Mazzini, Giacomo P. Comi, Roberto Del Bo, Mauro Ceroni, Stella Gagliardi, Giorgia Querin, Cinzia Bertolin, Wouter van Rheenen, Rosa Rademakers, Marka van Blitterswijk, Giuseppe Lauria, Stefano Duga, Stefania Corti, Cristina Cereda, Lucia Corrado, Gianni Sorarù, Kelly L. Williams, Garth A. Nicholson, Ian P. Blair, Claire Leblond-Manry, Guy A. Rouleau, Orla Hardiman, Karen E. Morrison, Jan H. Veldink, Leonard H. van den Berg, Ammar Al-Chalabi, Hardev Pall, Pamela J. Shaw, Martin R. Turner, Kevin Talbot, Franco Taroni, Alberto García-Redondo, Zheyang Wu, Jonathan D. Glass, Cinzia Gellera, Antonia Ratti, Robert H. Brown, Vincenzo Silani, Christopher E. Shaw, John E. Landers, Clifton L. Dalgard, Adelani Adeleye, Anthony R. Soltis, Camille Alba, Coralie Viollet, Dagmar Bacikova, Daniel N. Hupalo, Gauthaman Sukumar, Harvey B. Pollard, Matthew D. Wilkerson, Elisa McGrath Martinez, Yevgeniya Abramzon, Sarah Ahmed, Sampath Arepalli, Robert H. Baloh, Robert Bowser, Christopher B. Brady, Alexis Brice, James Broach, Roy H. Campbell, William Camu, Ruth Chia, John Cooper-Knock, Jinhui Ding, Carsten Drepper, Vivian E. Drory, Travis L. Dunckley, John D. Eicher, Bryce K. England, Faraz Faghri, Eva Feldman, Mary Kay Floeter, Pietro Fratta, Joshua T. Geiger, Glenn Gerhard, J. Raphael Gibbs, Summer B. Gibson, Jonathan D. Glass, John Hardy, Matthew B. Harms, Terry D. Heiman-Patterson, Dena G. Hernandez, Lilja Jansson, Janine Kirby, Neil W. Kowall, Hannu Laaksovirta, Natalie Landeck, Francesco Landi, Isabelle Le Ber, Serge Lumbroso, Daniel J. L. MacGowan, Nicholas J. Maragakis, Gabriele Mora, Kevin Mouzat, Natalie A. Murphy, Liisa Myllykangas, Mike A. Nalls, Richard W. Orrell, Lyle W. Ostrow, Roger Pamphlett, Stuart Pickering-Brown, Erik P. Pioro, Olga Pletnikova, Hannah A. Pliner, Stefan M. Pulst, John M. Ravits, Alan E. Renton, Alberto Rivera, Wim Robberecht, Ekaterina Rogaeva, Sara Rollinson, Jeffrey D. Rothstein, Sonja W. Scholz, Michael Sendtner, Pamela J. Shaw, Katie C. Sidle, Zachary Simmons, Andrew B. Singleton, Nathan Smith, David J. Stone, Pentti J. Tienari, Juan C. Troncoso, Miko Valori, Philip Van Damme, Vivianna M. Van Deerlin, Ludo Van Den Bosch, Lorne Zinman, John E. Landers, Adriano Chiò, Bryan J. Traynor, Stefania M. Angelocola, Francesco P. Ausiello, Marco Barberis, Ilaria Bartolomei, Stefania Battistini, Enrica Bersano, Giulia Bisogni, Giuseppe Borghero, Maura Brunetti, Corrado Cabona, Andrea Calvo, Fabrizio Canale, Antonio Canosa, Teresa A. Cantisani, Margherita Capasso, Claudia Caponnetto, Patrizio Cardinali, Paola Carrera, Federico Casale, Adriano Chiò, Tiziana Colletti, Francesca L. Conforti, Amelia Conte, Elisa Conti, Massimo Corbo, Stefania Cuccu, Eleonora Dalla Bella, Eustachio D’Errico, Giovanni DeMarco, Raffaele Dubbioso, Carlo Ferrarese, Pilar M. Ferraro, Massimo Filippi, Nicola Fini, Gianluca Floris, Giuseppe Fuda, Salvatore Gallone, Giulia Gianferrari, Fabio Giannini, Maurizio Grassano, Lucia Greco, Barbara Iazzolino, Alessandro Introna, Vincenzo La Bella, Serena Lattante, Giuseppe Lauria, Rocco Liguori, Giancarlo Logroscino, Francesco O. Logullo, Christian Lunetta, Paola Mandich, Jessica Mandrioli, Umberto Manera, Fiore Manganelli, Giuseppe Marangi, Kalliopi Marinou, Maria Giovanna Marrosu, Ilaria Martinelli, Sonia Messina, Cristina Moglia, Gabriele Mora, Lorena Mosca, Maria R. Murru, Paola Origone, Carla Passaniti, Cristina Petrelli, Antonio Petrucci, Susanna Pozzi, Maura Pugliatti, Angelo Quattrini, Claudia Ricci, Giulia Riolo, Nilo Riva, Massimo Russo, Mario Sabatelli, Paolina Salamone, Marco Salivetto, Fabrizio Salvi, Marialuisa Santarelli, Luca Sbaiz, Riccardo Sideri, Isabella Simone, Cecilia Simonini, Rossella Spataro, Raffaella Tanel, Gioacchino Tedeschi, Anna Ticca, Antonella Torriello, Stefania Tranquilli, Lucio Tremolizzo, Francesca Trojsi, Rosario Vasta, Veria Vacchiano, Giuseppe Vita, Paolo Volanti, Marcella Zollino, Elisabetta Zucchi

**Affiliations:** 1 Neuromuscular Diseases Research Section, Laboratory of Neurogenetics, National Institute on Aging, National Institutes of Health, Bethesda, Maryland; 2 Division of Medical Genetics, Department of Medicine, University of Washington, Seattle; 3 Department of Pediatrics, Division of Genetic Medicine, Seattle Children’s Hospital, University of Washington, Seattle; 4 Department of Pediatrics, Children’s Hospital of Richmond at VCU, Richmond, Virginia; 5 Cell Biology and Gene Expression Section, Laboratory of Neurogenetics, National Institute on Aging, National Institutes of Health, Bethesda, Maryland; 6 Reta Lila Weston Institute, UCL Queen Square Institute of Neurology, University College London, London, United Kingdom; 7 Maurice Wohl Clinical Neuroscience Institute, Institute of Psychiatry, Psychology and Neuroscience, King’s College London, London, United Kingdom; 8 Department of Biology, Imam Abdulrahman bin Faisal University, Dammam, Saudi Arabia; 9 Nash Family Department of Neuroscience, Icahn School of Medicine at Mount Sinai, New York, New York; 10 Ronald M. Loeb Center for Alzheimer’s Disease, Icahn School of Medicine at Mount Sinai, New York, New York; 11 Department of Genetics and Genomic Sciences, Icahn School of Medicine at Mount Sinai, New York, New York; 12 UK Dementia Research Institute at King’s College London, London, United Kingdom; 13 Computational Biology Group, Laboratory of Neurogenetics, National Institute on Aging, National Institutes of Health, Bethesda, Maryland; 14 Neurodegenerative Diseases Research Unit, Laboratory of Neurogenetics, National Institute of Neurological Disorders and Stroke, National Institutes of Health, Bethesda, Maryland; 15 Department of Anatomy, Physiology & Genetics, Uniformed Services University of the Health Sciences, Bethesda, Maryland; 16 The American Genome Center, Collaborative Health Initiative Research Program, Uniformed Services University of the Health Sciences, Bethesda, Maryland; 17 Istituto Auxologico Italiano, IRCCS, Department of Neurology–Stroke Unit and Laboratory of Neuroscience, Milan, Italy; 18 Department of Pathophysiology and Transplantation, “Dino Ferrari” Center, Università degli Studi di Milano, Milan, Italy; 19 Unit of Genetics of Neurodegenerative and Metabolic Diseases, Fondazione IRCCS Istituto Neurologico ‘Carlo Besta,’ Milan, Italy; 20 Centre for Motor Neuron Disease Research, Department of Biomedical Sciences, Faculty of Medicine and Health Sciences, Macquarie University, Sydney, Australia; 21 The University of Sydney, Brain and Mind Centre and School of Medical Sciences, Faculty of Medicine and Health, Camperdown, Australia; 22 School of Medical Sciences, University of New South Wales, Kensington, Australia; 23 Suna and Inan Kırac Foundation, Neurodegeneration Research Laboratory, KUTTAM, School of Medicine, Koc University, Istanbul, Turkey; 24 Department of Neurology, Helsinki University Hospital and Translational Immunology Programme, Biomedicum, University of Helsinki, Helsinki, Finland; 25 Faculty of Medicine, Health and Life Sciences, Queen’s University Belfast, Belfast, United Kingdom; 26 Sheffield Institute for Translational Neuroscience, Department of Neuroscience, University of Sheffield, Sheffield, United Kingdom; 27 Department of Neurology, King’s College Hospital, London, United Kingdom; 28 Department of Neurology, University of Massachusetts Medical School, Worcester; 29 ALS Center, ‘Rita Levi Montalcini’ Department of Neuroscience, University of Turin, Turin, Italy; 30 ALS Center, ICS Maugeri, IRCCS, Milan, Italy; 31 Department of Neurology, Children’s Hospital of Richmond at VCU, Richmond, Virginia; 32 Department of Neurology, The Agnes Ginges Center for Human Neurogenetics, Hadassah Medical Organization and Faculty of Medicine, Hebrew University of Jerusalem, Jerusalem, Israel; 33 Department of Neurology, Seattle Children’s Hospital, University of Washington, Seattle; 34 Department of Laboratories, Seattle Children’s Hospital, Seattle, Washington; 35 Department of Laboratory Medicine, University of Washington, Seattle; 36 Neurology 1, AOU Città della Salute e della Scienza, Turin, Italy; 37 Department of Neurology, Johns Hopkins University, Baltimore, Maryland; 38 Department of Pediatrics, Johns Hopkins University, Baltimore, Maryland; 39 National Institute of Neurological Disorders and Stroke, Bethesda, Maryland; 40 Centre for Neurodegeneration Research, King’s College London, Department of Clinical Neuroscience, Institute of Psychiatry, London, United Kingdom; 41 Department of Neurology and Laboratory of Neuroscience, IRCCS Istituto Auxologico Italiano, Milan, Italy; 42 Department of Pathophysiology and Transplantation, “Dino Ferrari” Center, Università degli Studi di Milano, Milan, Italy; 43 Department of Neurology, University of Massachusetts Medical School, Worcester; 44 UK Dementia Research Institute at King’s College London, London, United Kingdom; 45 Centre for Brain Research, University of Auckland, Auckland, New Zealand; 46 Department of Neurology, IRCCS Istituto Auxologico Italiano, Milan, Italy; 47 Maurice Wohl Clinical Neuroscience Institute, Department of Basic and Clinical Neuroscience, King’s College London, London, United Kingdom; 48 Unit of Medical Genetics and Neurogenetics, Fondazione IRCCS Istituto Neurologico ‘Carlo Besta,’ Milan, Italy; 49 Clinical Genetics, Leiden University Medical Center, Leiden, the Netherlands; 50 Department of Neurogenetics and Neurology, Academic Medical Centre, Amsterdam, the Netherlands; 51 Population Genetics Laboratory, Smurfit Institute of Genetics, Trinity College Dublin, Dublin, Republic of Ireland; 52 Department of Neurology, Emory University, Atlanta, Georgia; 53 Unidad de ELA, Instituto de Investigación Hospital 12 de Octubre de Madrid, SERMAS, and Centro de Investigación Biomédica en Red de Enfermedades Raras (CIBERER U-723), Madrid, Spain; 54 Neurology Clinic, Clinical Center of Serbia School of Medicine, University of Belgrade, Belgrade, Serbia; 55 Department of Health Sciences, University of Eastern Piedmont, Novara, Italy; 56 ALS Center, Azienda Ospedaliero Universitaria Maggiore della Carità, Novara, Italy; 57 Neurology Unit, IRCCS Foundation Ca’ Granda Ospedale Maggiore Policlinico, Milan, Italy; 58 Department of Brain and Behavior, University of Pavia, Pavia, Italy; 59 General Neurology Unit, IRCCS Mondino Foundation, Pavia, Italy; 60 Genomic and Post-Genomic Center, Mondino Foundation—IRCCS, Pavia, Italy; 61 Department of Neurosciences, University of Padova, Padova, Italy; 62 Department of Neurology, Brain Center Rudolf Magnus, University Medical Center Utrecht, Utrecht, the Netherlands; 63 Department of Neuroscience, Mayo Clinic, Jacksonville, Florida; 64 3rd Neurology Unit, Motor Neuron Diseases Center, Fondazione IRCCS Istituto Neurologico ‘Carlo Besta,’ Milan, Italy; 65 Humanitas Clinical and Research Center–IRCCS, Milan, Italy; 66 Department of Biomedical Sciences, Humanitas University, Milan, Italy; 67 Centre for Motor Neuron Disease Research, Department of Biomedical Sciences, Faculty of Medicine and Health Sciences, Macquarie University, Sydney, Australia; 68 Molecular Medicine Laboratory, Clinical Sciences Building, Concord Hospital, Concord, Australia; 69 Human Genetics and Cognitive Functions Unit, Institut Pasteur, Paris, France; 70 Montreal Neurological Institute, Department of Neurology and Neurosurgery, McGill University, Montreal, Quebec, Canada; 71 Academic Unit of Neurology, Trinity Biomedical Sciences Institute, Trinity College Dublin, Dublin, Republic of Ireland; 72 Faculty of Medicine, Health and Life Sciences, Queen’s University Belfast, Belfast, United Kingdom; 73 School of Clinical and Experimental Medicine, University of Birmingham, Birmingham, United Kingdom; 74 Sheffield Institute for Translational Neuroscience, Department of Neuroscience, University of Sheffield, Sheffield, United Kingdom; 75 Nuffield Department of Clinical Neurosciences, University of Oxford, Oxford, United Kingdom; 76 Department of Bioinformatics and Computational Biology, Worcester Polytechnic Institute, Worcester, Massachusetts; 77 The American Genome Center, Collaborative Health Initiative Research Program, Uniformed Services University of the Health Sciences, Bethesda, Maryland; 78 Department of Anatomy, Physiology & Genetics, Uniformed Services University of the Health Sciences, Bethesda, Maryland; 79 Henry M. Jackson Foundation for the Advancement of Military Medicine, Bethesda, Maryland; 80 Neuromuscular Diseases Research Section, Laboratory of Neurogenetics, National Institute on Aging, Bethesda, Maryland; 81 Sobell Department of Motor Neuroscience and Movement Disorders, Institute of Neurology, University College London, London, United Kingdom; 82 Neurodegenerative Diseases Research Unit, Laboratory of Neurogenetics, National Institute of Neurological Disorders and Stroke, Bethesda, Maryland; 83 Genomics Technology Group, Laboratory of Neurogenetics, National Institute on Aging, Bethesda, Maryland; 84 Department of Neurology, Cedars-Sinai Medical Center, Los Angeles, California; 85 Division of Neurology, Barrow Neurological Institute, Phoenix, Arizona; 86 Research and Development Service, Veterans Affairs Boston Healthcare System, Boston, Massachusetts; 87 Centre de Recherche de l’Institut du Cerveau et de la Moelle épinière, Université Pierre et Marie Curie, Paris, France; 88 INSERM U975, Paris, France; 89 Department of Biochemistry, Penn State College of Medicine, Hershey, Pennsylvania; 90 Department of Computer Science, University of Illinois at Urbana-Champaign, Urbana; 91 ALS Reference Center, Gui de Chauliac Hospital, CHU and University of Montpellier, Montpellier, France; 92 Department of Neuroscience, University of Sheffield, Sheffield, United Kingdom; 93 Computational Biology Group, Laboratory of Neurogenetics, National Institute on Aging, Bethesda, Maryland; 94 Institute for Clinical Neurobiology, University of Würzburg, Würzburg, Germany; 95 Department of Neurology, Tel-Aviv Sourasky Medical Center, Tel-Aviv, Israel; 96 Neurodegenerative Disease Research Center, Biodesign Institute, Arizona State University, Tempe; 97 Department of Genetics and Pharmacogenomics, Merck Research Laboratories, Merck, West Point, Pennsylvania; 98 Human Genetics Branch, National Institute of Mental Health, Bethesda, Maryland; 99 Molecular Genetics Section, Laboratory of Neurogenetics, National Institute on Aging, Bethesda, Maryland; 100 Department of Neurology, University of Michigan, Ann Arbor; 101 Motor Neuron Disorders Unit, Laboratory of Neurogenetics, National Institute of Neurological Disorders and Stroke, Bethesda, Maryland; 102 Department of Pathology, Penn State College of Medicine, Hershey, Pennsylvania; 103 Department of Neurology, University of Utah School of Medicine, Salt Lake City; 104 Department of Neurology, Emory University School of Medicine, Atlanta, Georgia; 105 Department of Molecular Neuroscience and Reta Lila Weston Laboratories, Institute of Neurology, University College London, London, United Kingdom; 106 Department of Neurology, Columbia University, New York, New York; 107 Department of Neurology, Drexel University College of Medicine, Philadelphia, Pennsylvania; 108 Department of Neurology, Temple University, Philadelphia, Pennsylvania; 109 Department of Neurology, University of Helsinki, Helsinki, Finland; 110 Department of Neurology, Veterans Affairs Boston Healthcare System, Boston, Massachusetts; 111 Cell Biology and Gene Expression Section, Laboratory of Neurogenetics, National Institute on Aging, Bethesda, Maryland; 112 Department of Geriatrics, Neurosciences and Orthopedics, Center for Geriatric Medicine, Catholic University of Sacred Heart, Rome, Italy; 113 INM, INSERM, University of Montpellier, CHU Nîmes, Nîmes, France; 114 Neuromuscular Division and ALS Center, Beth Israel Medical Center, Albert Einstein College of Medicine, New York, New York; 115 Department of Neurology, Johns Hopkins University, Baltimore, Maryland; 116 ALS Center, ICS Maugeri, IRCCS, Milan, Italy; 117 Department of Pathology, Haartman Institute/HUSLAB, University of Helsinki and Folkhalsan Research Center (LM), Helsinki, Finland; 118 Data Tecnica International, Glen Echo, Maryland; 119 Department of Clinical Neuroscience, Institute of Neurology, University College London, London, United Kingdom; 120 Discipline of Pathology, Brain and Mind Centre, University of Sydney, Camperdown, Australia; 121 Faculty of Human and Medical Sciences, University of Manchester, Manchester, United Kingdom; 122 Department of Neurology, Cleveland Clinic, Cleveland, Ohio; 123 Clinical and Neuropathology Core, Johns Hopkins University, Baltimore, Maryland; 124 Department of Neuroscience, Experimental Neurology and Leuven Research Institute for Neuroscience and Disease, University of California San Diego, La Jolla; 125 Nash Family Department of Neuroscience, Ronald M. Loeb Center for Alzheimer’s Disease, Icahn School of Medicine at Mount Sinai, New York, New York; 126 Department of Neurosciences, Experimental Neurology and Leuven Research Institute for Neuroscience and Disease, University of Leuven, Leuven, Belgium; 127 Tanz Centre for Research of Neurodegenerative Diseases, University of Toronto, Toronto, Ontario, Canada; 128 Department of Neurology, Institute for Clinical Neurobiology, University of Würzburg, Würzburg, Germany; 129 Department of Neurology, Penn State College of Medicine, Hershey, Pennsylvania; 130 VIB, Center for Brain & Disease Research, Laboratory of Neurobiology, University of Leuven, Leuven, Belgium; 131 Department of Pathology and Laboratory Medicine, University of Pennsylvania, Philadelphia; 132 Division of Neurology, Sunnybrook Health Sciences Centre, University of Toronto, Toronto, Ontario, Canada; 133 Department of Neurology, University of Massachusetts Medical School, Worcester; 134 ‘Rita Levi Montalcini’ Department of Neuroscience, University of Turin, Turin, Italy; 135 Neuroscience Institute of Torino, University of Turin, Turin, Italy; 136 National Institute of Neurological Disorders and Stroke, Bethesda, Maryland; 137 Neurology Unit, AV4 ASUR Marche, Fermo, Italy; 138 Department of Medical Genetic, Azienda Ospedaliero Universitaria Città della Salute e della Scienza, Torino, Italy; 139 Center for Diagnosis and Cure of Rare Diseases, Department of Neurology, IRCCS Institute of Neurological Sciences, Bologna, Italy; 140 Department of Medical, Surgical and Neurological Sciences, University of Siena, Siena, Italy; 141 3rd Neurology Unit and Motor Neuron Diseases Centre, IRCCS Foundation “Carlo Besta” Neurological Institute, Milan, Italy; 142 NeuroMuscular Omnicentre (NEMO), Serena Onlus, Foundation Pol. A. Gemelli, Roma, Italy; 143 Neurologic Unit, Monserrato University Hospital, Cagliari University, Cagliari, Italy; 144 Department of Neurosciences, Rehabilitation, Ophthalmology, Genetics and Maternal-Child Sciences, IRCCS Ospedale Policlinico San Martino, Genoa, Italy; 145 Division of Neurology, Azienda Ospedaliero–Universitaria Città della Salute e della Scienza di Torino, Torino, Italy; 146 Department of Advanced Medical and Surgical Sciences, University of Campania “Luigi Vanvitelli,” Caserta, Italy; 147 Struttura complessa di Neurofisiopatologia, Azienda Ospedaliera di Perugia, Perugia, Italy; 148 Unit of Neurology, Ospedale Clinicizzato SS Annunziata, Chieti, Italy; 149 Unit of Genomics for the Diagnosis of Human Pathologies, IRCCS San Raffaele Scientific Institute, Milan, Italy; 150 ALS Clinical Research Center, BiND, University of Palermo, Palermo, Italy; 151 Department of Pharmacy, Health and Nutritional Sciences, University of Calabria, Rende, Italy; 152 Neurology Unit, “San Gerardo” Hospital, Monza, Italy; 153 School of Medicine and Surgery and Milan Center for Neuroscience (NeuroMI), University of Milano-Bicocca, Milan, Italy; 154 Department of Neurorehabilitation Sciences, Casa Cura Policlinico, Milan, Italy; 155 Department of Basic Medical Sciences, Neurosciences and Sense Organs, University of Bari “Aldo Moro,” Policlinic, Bari, Italy; 156 Department of Neurosciences, Reproductive Sciences and Odontostomatology, University of Naples Federico II, Napoli, Italy; 157 Neurology Unit and Rehabilitation Unit, IRCCS “San Raffaele Scientific Institute,” Milan, Italy; 158 Neuroimaging Research Unit, Division of Neuroscience, IRCCS “San Raffaele Scientific Institute,” Milan, Italy; 159 Neurophysiology Service, IRCCS “San Raffaele Scientific Institute,” Milan, Italy; 160 Vita-Salute San Raffaele University, Milan, Italy; 161 Department of Neurosciences, Ospedale Civile S. Agostino Estense, Azienda Ospedaliero Universitaria di Modena, Modena, Italy; 162 NeuroMuscular Omnicentre (NEMO) Clinical Center Milano, Fondazione Serena Onlus, Milan, Italy; 163 Genetica Medica, Fondazione Policlinico Universitario A. Gemelli IRCCS, Roma, Italy; 164 Dipartimento Universitario Scienze della Vita e Sanità Pubblica, Sezione di Medicina Genomica, Università Cattolica del Sacro Cuore Facoltà di Medicina e Chirurgia, Roma, Italy; 165 Department of Biomedical and Clinical Sciences Luigi Sacco, University of Milan, Milan, Italy; 166 Department of Biomedical and Neuromotor Sciences, University of Bologna, Bologna, Italy; 167 Department of Basic Medical Sciences, Neuroscience and Sense Organs, University of Bari Aldo Moro, Bari, Italy; 168 Center for Neurodegenerative Diseases and the Aging Brain, Department of Clinical Research in Neurology, Pia Fondazione Cardinale G. Panico, Tricase, Italy; 169 Neurology Unit, AV3, ASUR Marche, Macerata, Italy; 170 Medical Genetics Unit, IRCCS Ospedale Policlinico San Martino, Genoa, Italy; 171 Department of Neurorehabilitation, Istituti Clinici Scientifici Maugeri IRCCS, Institute of Milan, Milan, Italy; 172 Department of Neurology, Azienda Universitario Ospedaliera di Cagliari and University of Cagliari, Cagliari, Italy; 173 NeuroMuscular Omnicentre (NEMO) Sud, Fondazione Aurora, OUC Neurology and Neuromuscular Disorders, University of Messina, Messina, Italy; 174 Department of Laboratory Medicine, Medical Genetics, Niguarda Ca’ Granda Hospital, Milan, Italy; 175 Department of Advanced Medical and Surgical Sciences, University of Campania “Luigi Vanvitelli,” Napoli, Italy; 176 Neurology Department, San Camillo Hospital, Roma, Italy; 177 Department of Biomedical and Surgical Sciences, Section of Neurological, Psychiatric and Psychological Sciences, University of Ferrara, Ferrara, Italy; 178 Department of Neurology, IRCCS “San Raffaele Scientific Institute,” Milan, Italy; 179 OUC Neurology and Neuromuscular Disorders, University of Messina, Messina, Italy; 180 NeuroMuscular Omnicentre (NEMO)–Fondazione Policlinico Universitario A. Gemelli, Università Cattolica del Sacro Cuore, Roma, Italy; 181 Department of Medicine, Azienda Complesso Ospedaliero, San Filippo Neri, Roma, Italy; 182 Operative Unit of Neurology, S. Chiara Hospital, Trento, Italy; 183 Department of Neurology, Azienda Ospedaliera San Francesco, Nuoro, Italy; 184 ALS Center, Operative Unit of Neurology, AOU “San Giovanni di Dio e Ruggi d’Aragona,” Salerno, Italy; 185 Neurorehabilitation Unit–ALS Center, Istituti Clinici Scientifici (ICS) Maugeri, Mistretta, Italy; 186 Sezione di Medicina Genomica, Dipartimento Scienze della Vita e Sanità Pubblica, Facoltà di Medicina e Chirurgia, Università Cattolica Sacro Cuore, Roma, Italy; 187 Unità di Genetica Medica, Fondazione Policlinico Universitario A. Gemelli IRCCS, Roma, Italy

## Abstract

**Importance:**

Juvenile amyotrophic lateral sclerosis (ALS) is a rare form of ALS characterized by age of symptom onset less than 25 years and a variable presentation.

**Objective:**

To identify the genetic variants associated with juvenile ALS.

**Design, Setting, and Participants:**

In this multicenter family-based genetic study, trio whole-exome sequencing was performed to identify the disease-associated gene in a case series of unrelated patients diagnosed with juvenile ALS and severe growth retardation. The patients and their family members were enrolled at academic hospitals and a government research facility between March 1, 2016, and March 13, 2020, and were observed until October 1, 2020. Whole-exome sequencing was also performed in a series of patients with juvenile ALS. A total of 66 patients with juvenile ALS and 6258 adult patients with ALS participated in the study. Patients were selected for the study based on their diagnosis, and all eligible participants were enrolled in the study. None of the participants had a family history of neurological disorders, suggesting de novo variants as the underlying genetic mechanism.

**Main Outcomes and Measures:**

De novo variants present only in the index case and not in unaffected family members.

**Results:**

Trio whole-exome sequencing was performed in 3 patients diagnosed with juvenile ALS and their parents. An additional 63 patients with juvenile ALS and 6258 adult patients with ALS were subsequently screened for variants in the *SPTLC1* gene. De novo variants in *SPTLC1* (p.Ala20Ser in 2 patients and p.Ser331Tyr in 1 patient) were identified in 3 unrelated patients diagnosed with juvenile ALS and failure to thrive. A fourth variant (p.Leu39del) was identified in a patient with juvenile ALS where parental DNA was unavailable. Variants in this gene have been previously shown to be associated with autosomal-dominant hereditary sensory autonomic neuropathy, type 1A, by disrupting an essential enzyme complex in the sphingolipid synthesis pathway.

**Conclusions and Relevance:**

These data broaden the phenotype associated with *SPTLC1* and suggest that patients presenting with juvenile ALS should be screened for variants in this gene.

## Key Points

**Question:**

What genetic variants are associated with juvenile amyotrophic lateral sclerosis (ALS)?

**Findings:**

In this family-based genetic study, exome sequencing was performed in 3 patients diagnosed with juvenile ALS and failure to thrive; this identified de novo variants in *SPTLC1* (p.Ala20Ser in 2 patients and p.Ser331Tyr in 1 patient). Variants in *SPTLC1* are a known cause of hereditary sensory and autonomic neuropathy, type 1A, and these data extend the phenotype associated with this gene.

**Meaning:**

De novo variants in the *SPTLC1* gene are associated with juvenile ALS, a fatal neurological disorder.

## Teaser

This genetic association study identifies genetic variants associated with juvenile amyotrophic lateral sclerosis.

## Introduction

Amyotrophic lateral sclerosis (ALS) is a relatively common neurological disorder characterized by progressive paralysis and death from respiratory failure. The vast majority of cases occur in individuals older than 40 years.^1^ In contrast, juvenile ALS (defined as an age of onset less than 25 years) is a rare form of motor neuron disease.^2^ These early-onset cases are characterized by slow progression and a variable phenotype that often makes accurate diagnosis challenging.^2^

Considerable progress has been made in unravelling the genetic architecture underlying ALS, but much remains to be understood about this condition.^3^ Juvenile ALS is thought to be more frequently genetic in origin than the adult-onset forms, and the genetic analysis of these young-onset cases offers an opportunity to identify disease-causing genes.^2^ By extension, any gene underlying juvenile ALS may also play a role in adult-onset ALS.

De novo genetic variants may underlie at least a portion of ALS cases. Such variants would not be detected by genome-wide association studies owing to their recent occurrence and corresponding low frequency within the community. Spontaneously occurring variants are a well-known cause of neurological conditions, such as neurofibromatosis type 1 and Duchenne muscular dystrophy.^4,5^ Indeed, de novo variants of the familial ALS genes *FUS*, *SOD1*, and *VCP* have been described in sporadic ALS cases.^6,7^ Such variants are more likely to present with early-onset disorders because of their impact on fitness.^8^

Here, we performed whole-exome sequencing of 3 patients diagnosed with juvenile ALS and their unaffected parents to identify the variants associated with their disease. None of these patients had a family history of neuromuscular disorders, suggesting de novo variations as the underlying genetic mechanism. After identifying variants in serine palmitoyltransferase, long-chain base subunit 1 (*SPTLC1*; OMIM, 605712) in all 3 cases, we also screened patients with juvenile ALS from Turkey for *SPTLC1* variants and identified a fourth patient carrying an *SPTLC1* variant.

## Methods

### Patients

Four unrelated patients with neuromuscular symptoms consistent with juvenile ALS participated in the study between March 2016 and January 2021. The Table summarizes the clinical features of the 4 patients. Detailed descriptions of each patient and their recruitment are available in the eMethods in the Supplement. All participants provided written informed consent for genetic analysis according to the Declaration of Helsinki, and the Institutional Review Board of the National Institutes of Health approved the study. Members of the FALS Sequencing Consortium, American Genome Center, International ALS Genomics Consortium, and ITALSGEN Consortium can be found in the eAppendix of the Supplement.

**Table.** Clinical Features of Patients Diagnosed With Juvenile Amyotrophic Lateral Sclerosis and Carrying Variants in *SPTLC1*

Clinical feature	Patient 1	Patient 2	Patient 3	Patient 4
Gene change	p.Ala20Ser	p.Ala20Ser^**a**^	p.Ser331Tyr	p.Leu39del
Age at onset	5 y	<10 y	4 y	15 y
Age at evaluation	20 y	10s	11 y	34 y
BMI (*z* score)	13th Percentile (−1.1)	<1st Percentile (−7.0)	<1st Percentile (−6.5)	Normal
Back deformities	Severe scoliosis	Lordosis	Normal posture	NA
Foot deformities	Pes cavus	NA	Pes cavus/varus	NA
Walking	Nonambulatory	Steppage	Steppage	Abnormal
Atrophy	Global, contractures	Global	Global	Global
Weakness	Generalized	Generalized	Generalized	Generalized
Reflexes	Hyporeflexia, Achilles tendon brisk	Hyporeflexia, Achilles tendon brisk	Hyperreflexia, Achilles tendon absent	Hyporeflexia
Tongue	Wasted, fasciculations	Wasted, fasciculations	Wasted, fasciculations	NA
Jaw jerk	Present	NA	Present	NA
Respiratory	Tracheostomy at 17 y	NA	Dyspnea on exercise	Normal
Cognition	Executive dysfunction	Executive dysfunction	Normal	NA
Sensory	Normal	Normal	Glove-stocking pain loss, foot ulceration	Normal
Neurophysiology				
Motor	Chronic denervation	Acute and chronic denervation	Axonal loss, polyphasia	Denerv
Sensory	Normal	Normal	Axonal loss	Normal
Additional features	NA	Scapular winging, Gower sign	Gower sign, vitamin D deficiency, hyperhidrosis	Uses a wheelchair

Abbreviations: BMI, body mass index; NA, not applicable.

^a^ Variant was detected in 1 of 49 and 0 of 149 next-generation sequencing reads from the father’s saliva-derived and buccal-derived DNA, respectively.

Patient 1 presented with gradually progressive spastic diplegia and growth retardation beginning at age 5 years. By age 20 years, she had quadriplegia with marked muscle atrophy and diminished weight, brisk lower limb reflexes, tongue fasciculations and weakness, dysarthria, mild cognitive dysfunction, and respiratory failure requiring tracheostomy and ventilation. Repeated neurophysiological testing did not show evidence of sensory or autonomic dysfunction. She was diagnosed with juvenile ALS based on the revised El Escorial criteria.^9^

**Figure 1.**
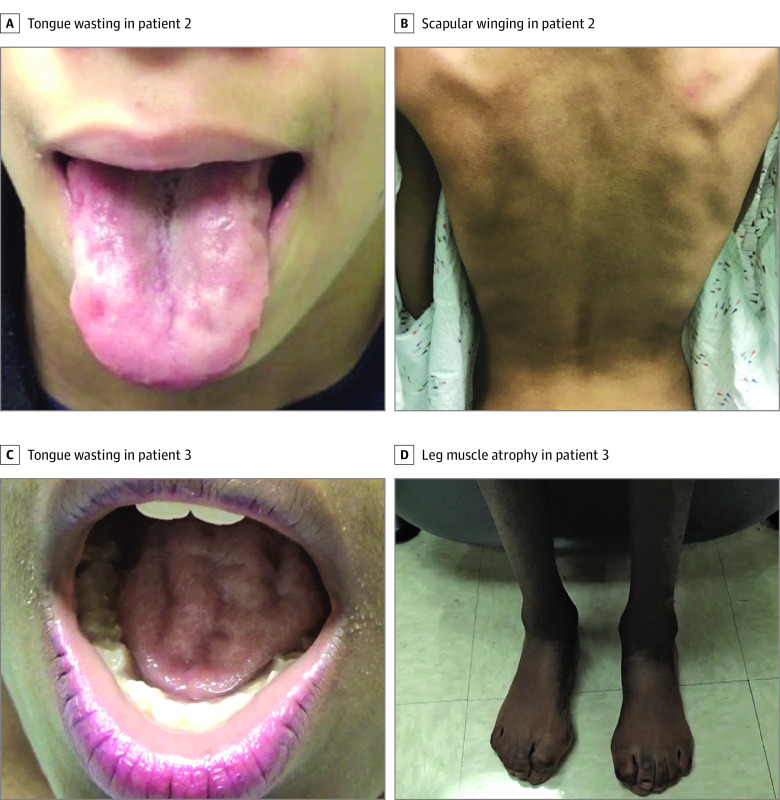
Clinical Features of Patients Diagnosed With Juvenile Amyotrophic Lateral Sclerosis A and B, Tongue wasting and scapular winging in patient 2 carrying the p.Ala20Ser *SPTLC1* variant. C and D, Tongue wasting and muscle atrophy of the lower limbs in patient 3 carrying the p.Ser331Tyr *SPTLC1* variant. Note the hammertoe deformities of both feet.

Patient 2 was a teenaged girl of African American and White race who presented with a 6-year history of gradually progressive generalized limb and bulbar weakness. She had a long-standing history of progressive weight loss of unknown cause, and her school performance began to decline in her mid-teens. Her neurological examination at presentation revealed a body mass index less than the first percentile, exaggerated lumbar lordosis, tongue fasciculations and wasting, generalized muscle atrophy and weakness, brisk asymmetric ankle reflexes, a positive Gower sign, and normal sensation (Figure 1A and B). Neurophysiological testing revealed active and chronic denervation without evidence of sensory neuropathy. Decreased sustained attention and impaired executive functioning were evident in neuropsychological evaluation. She was diagnosed with juvenile ALS based on the revised El Escorial criteria.^9^

**Figure 2.**
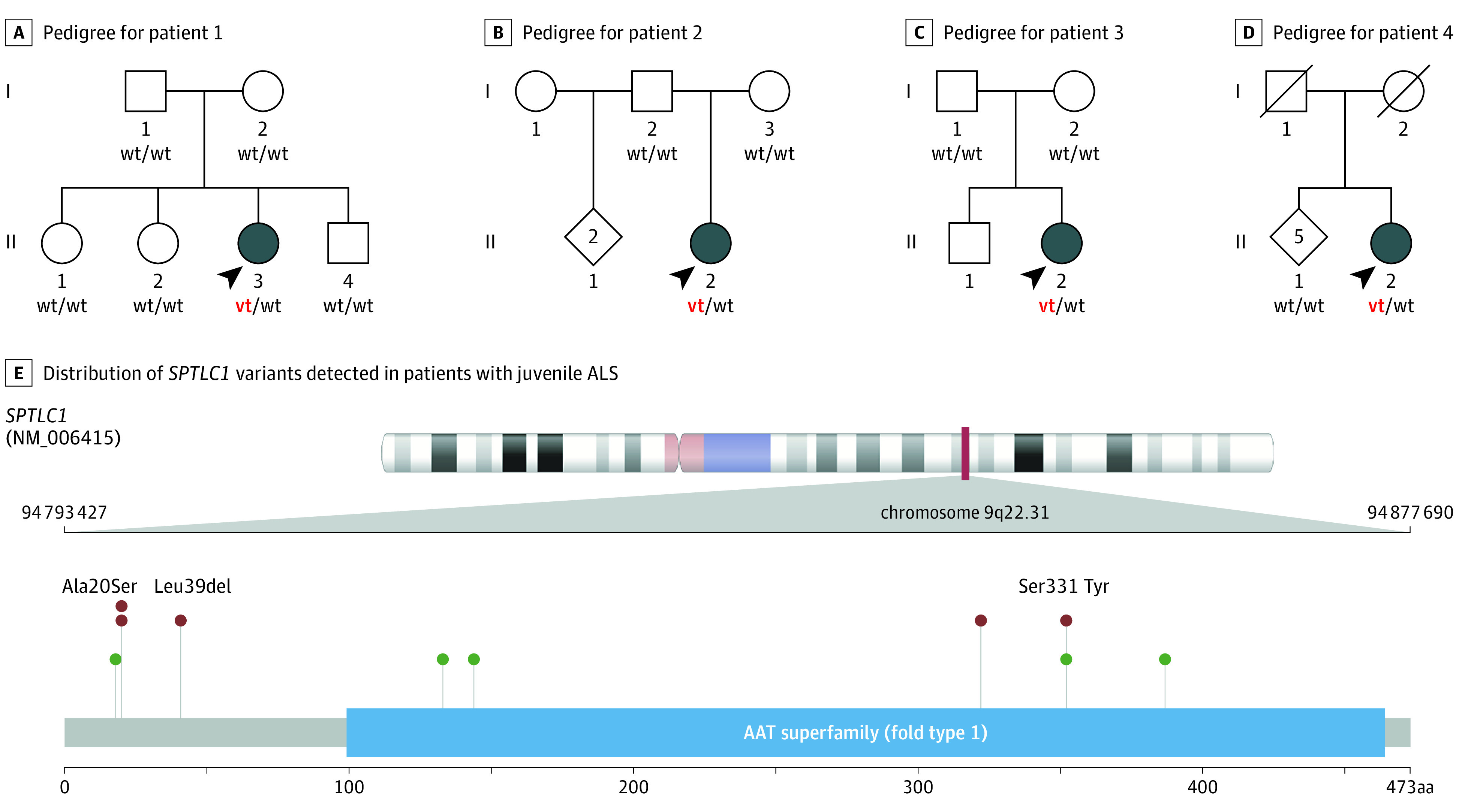
De Novo Variants of *SPTLC1* in Patients Diagnosed With Juvenile Amyotrophic Lateral Sclerosis (ALS) A-D, Pedigrees of 4 patients diagnosed with juvenile ALS. The variant alleles in *SPTLC1* are indicated by vt, and wild-type alleles are indicated by wt. The arrowheads indicate the probands. E, Distribution of *SPTLC1* variants detected in patients diagnosed with juvenile ALS. Variants identified in the 3 patients with juvenile ALS are noted in red, and variants previously described to cause hereditary sensory and autonomic neuropathy, type 1A, are shown in green.

Patient 3 was an 11-year-old African American girl with a history of failure to gain weight and toe walking since age 4 years. She presented at age 10 years with a deteriorating gait, hand weakness, right foot paresthesia, dysphagia, and increased sweating. Examination revealed marked atrophy, postural tachycardia, bilateral cataracts, a wasted and fasciculating tongue with an exaggerated jaw jerk, generalized fasciculations and weakness associated with hyperreflexia, and decreased pinprick sensation in a glove-and-stocking distribution (Figure 1C and D). The patient walked abnormally owing to weakness and bilateral foot drop, and she had a positive Gower sign. Neurophysiological examination showed sensorimotor axonal neuropathy as well as polyphasic potentials on electromyography. She was diagnosed with juvenile ALS-Plus syndrome owing to her prominent motor symptoms and modest sensory-autonomic involvement. The pedigrees of patients 1, 2, and 3 are shown in Figure 2A to C.

Patient 4 was a 34-year-old Turkish woman with a history of arm and leg weakness and atrophy since age 15 years. There was no family history of neuromuscular disease, and none of her 5 siblings had symptoms (Figure 2D). She was diagnosed with juvenile ALS, and she has been taking riluzole since age 15 years. Her symptoms were slowly progressive, and there were no upper motor neuron signs on examination. During her last review at age 34 years, she used a wheelchair, although she could walk short distances with assistance. She had no dysphagia and did not require oxygen supplementation, and her weight was normal. Neurophysiological examination at that time revealed denervation activity in all muscles and no evidence of multifocal motor neuropathy.

For variant screening of *SPTLC1* in adult-onset ALS, we used 6258 DNA samples obtained from individuals diagnosed with adult-onset ALS (eTable 1 in the Supplement). Control data consisted of 5710 neurologically healthy US individuals who had undergone next-generation sequencing at the Laboratory of Neurogenetics of the National Institute on Aging, National Institutes of Health, Bethesda, Maryland, or the Alzheimer Disease Sequencing Project.

### Next-Generation Sequencing in Juvenile ALS

Whole-exome sequencing was performed using 100 base-pair, paired-end sequencing on an Illumina sequencer (eg, HiSeq 2000) according to the manufacturer’s protocol. DNA from patient 1 and her family was sequenced in the Laboratory of Neurogenetics using TruSeq library preparation version 1.0. DNA from patients 2 and 3 and their families was sequenced at GeneDx using IDT xGen Exome Research Panel version 1.0.^10^

Data were analyzed to identify de novo variants present in the affected child and not present in either parent. As the variants underlying a rare disease, such as ALS, are unlikely to be present in the general population, variants present in the Genome Aggregation Database (gnomAD; version 2.1) or the Kaviar Genomic Variant database (September 23, 2015, version) were excluded. Synonymous, intronic, and intergenic changes were excluded (*ANNOVAR*; August 11, 2016, version). Paternity and maternity were confirmed using identity-by-descent analysis, and exome data were reviewed to identify variants in known ALS genes.

### *SPTLC1* Sequencing in Adult-Onset ALS

DNA from 6258 patients with adult-onset ALS were sequenced to identify variants in the *SPTLC1* gene (whole-exome sequencing, 3748 cases^11,12^; whole-genome sequencing, 1860 cases; Sanger sequencing, 650 cases) (eTable 2 in the Supplement). Variants in *SPTLC1* were considered to be deleterious if they (1) were not present in the 4647 controls from the Alzheimer Disease Sequencing Project; (2) had a frequency less than 3.3 × 10^−5^ in human variant databases, including the 51 592 European and 8949 Finnish nonneurological individuals in gnomAD and the 77 301 samples in Kaviar database^3^; and (3) were designated as damaging according to 4 of 5 prediction algorithms,^13^ were identified as stop gain or frameshift, or were identified as splice-site variants with a dbscSNV score higher than 0.6. Gene burden testing of *SPTLC1* was performed using publicly available control data (gnomAD and Kaviar databases) as implemented in the Test Rare Variants With Public Data (TRAPD) software package version 1.0.^14^ The statistical significance threshold was set at a 1-tailed *P *value less than .05 for single-gene analysis and 2.5 × 10^−6^ for genome-wide significance (.05/20 000 genes).

### Cellular Mitochondrial Assays

Variants were introduced into a plasmid containing the human *SPTLC1* open reading frame (Origene) using the QuikChange II XL kit (Agilent), followed by subcloning into pLenti-C-Myc-DDK-P2A-Puro lentiviral plasmid (Origene). Lentiviruses were produced with third-generation packaging plasmids (pMDLg/pRRE and pRSV-Rev; Addgene) and envelope plasmid (pMD2.G; Addgene).^15^ HEK293FT cells were transfected with wild-type or variant lentivirus transfer plasmid, and transduced cells stably expressing SPTLC1 were selected by extended growth in 0.5-μg/mL puromycin (Thermo Fisher Scientific). For the serine rescue experiment, a final concentration of 100-mM L-serine was added to the media for 48 hours. Mitochondria were imaged with MitoTracker Red CMXRos (Thermo Fisher Scientific) using a CellInsight imager (Thermo Fisher Scientific). A minimum of 6 wells were quantified for each condition, and all assays were performed at least twice. Unpaired *t* test with Welch correction or analysis of variance were used to calculate statistical significance.

### Sphingolipid Measurements

Plasma sphingolipid and glucosylceramide measurements were performed at the Biomedical Genetics Clinical Laboratory, Seattle Children’s Hospital, Seattle, Washington, using high-performance liquid chromatography and tandem mass spectrometry.

## Results

### Identification of De Novo Variants in *SPTLC1* Associated With Juvenile ALS

We performed whole-exome sequencing in 3 unrelated patients who had been diagnosed with juvenile ALS and their healthy parents (Figure 1; Figure 2A to C; Table). The analysis of their genetic data identified de novo variants in the *SPTLC1* gene in each of the 3 patients that were absent in their parents (Figure 2E). Patients 1 and 2 carried the same heterozygous p.Ala20Ser variant in *SPTLC1* caused by variation in adjacent nucleotides (chr9:94874844C>A and chr9:94874843G>T; human genome build hg19). Patient 3 carried a p.Ser331Tyr (chr9:94809543G>T) heterozygous variation in *SPTLC1*. Screening of the *SPTLC1* gene in a cohort of 63 patients with juvenile ALS from Turkey who had undergone whole-exome sequencing identified a p.Leu39del (chr9:94874785_94874787del) heterozygous variant in patient 4 (Figure 2D). Parental DNA was not available, making it impossible to determine if the deletion had occurred spontaneously. These *SPTLC1* variants were not present in controls or online databases of human variants (142 489 individuals). The p.Ser331Tyr and p.Leu39del amino acid changes have been previously implicated in neurological disease.^16^

### Serine and the Damaging Effects of the p.Ala20Ser Variant in Vitro

Variants in *SPTLC1* are a known cause of autosomal-dominant hereditary sensory and autonomic neuropathy, type 1A (HSAN1; OMIM, 162400).^17,18^ The protein encoded by *SPTLC1* is an essential subunit of serine palmitoyltransferase (SPT), the enzyme that catalyzes the first and rate-limiting step in the de novo synthesis of sphingolipids.^19^ A characteristic feature of *SPTLC1* variants associated with HSAN1 is a shift in substrate specificity of SPT to L-alanine and L-glycine, leading to the formation of an atypical class of deoxysphingolipids.^20^ These neurotoxic metabolites accumulate within cells as they cannot be converted to complex sphingolipids nor degraded by the catabolic pathway.^20^

**Figure 3.**
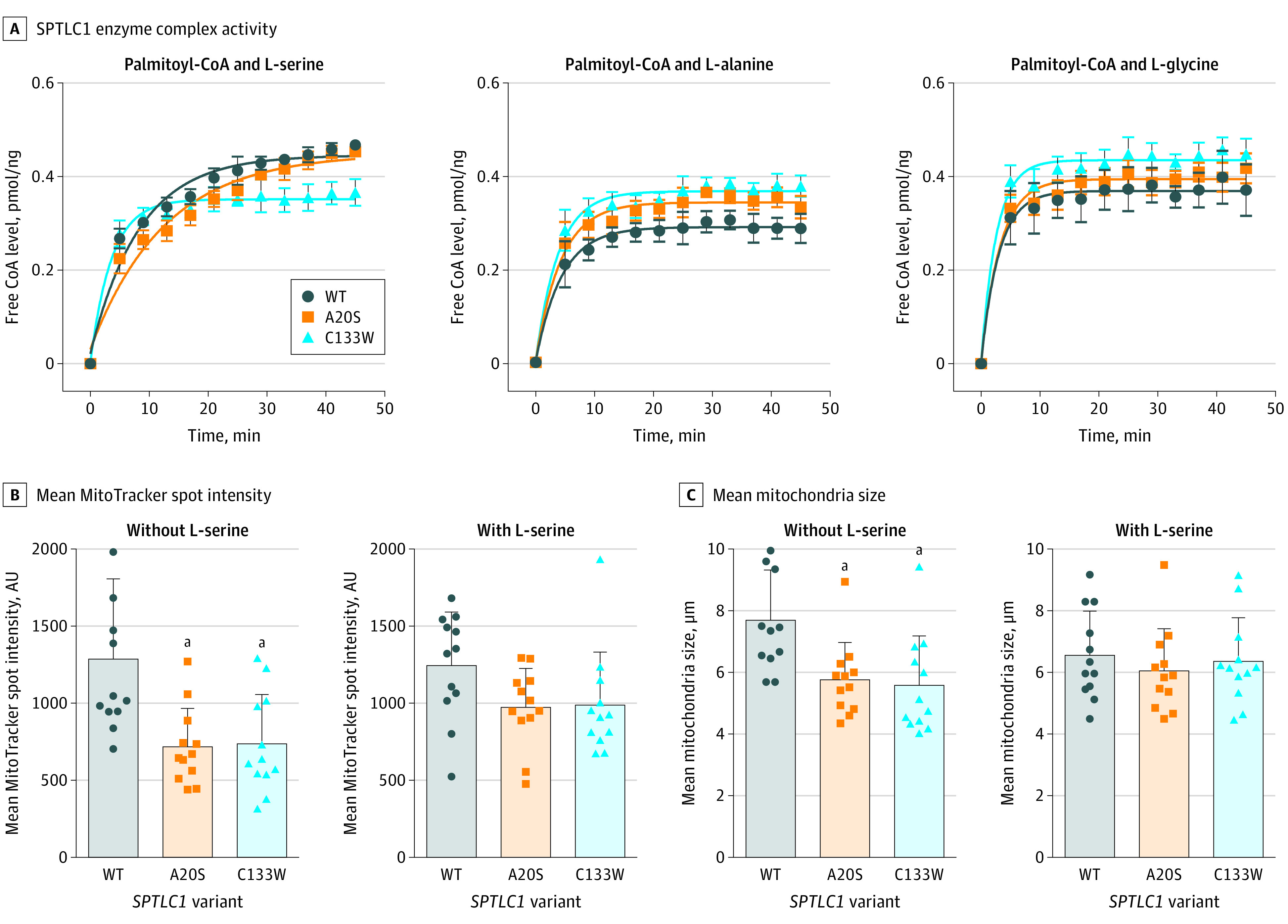
Photometric and Cell-Based Assays in the Presence of Select *SPTLC1* Variants A, The SPTLC1 enzyme complex activity was determined using a photometric assay measuring the release of free coenzyme A (coA) from the condensation reaction between palmitoyl-CoA and L-serine, L-alanine, and L-glycine. The variant p.Ala20Ser and p.Cys133Trp SPTLC1 complex had increased preference for L-alanine and L-glycine over L-serine compared with the wild-type (WT) SPTLC1 complex. B and C, Mitochondria in HEK293 cells expressing WT, p.Ala20Ser, and p.Cys133Trp were assessed using MitoTracker on a high-content imager. Mitochondrial intensity and mitochondria size were smaller in cells expressing variant protein under standard culture conditions. Supplementation of 100 mM L-serine in the culture media for 48 hours rescued the mitochondrial abnormalities in the p.Ala20Ser and p.Cys133Trp lines.

Based on this information, we used a photometric assay of SPT enzyme activity to explore the association of the de novo variants with protein function. We found that the p.Ala20Ser variant SPTLC1 complex had an altered L-alanine and glycine preference over the canonical L-serine compared with the wild-type SPTLC1 complex (Figure 3A; eFigure 1 in the Supplement). Differences were also observed using cell-based assays based on established HSAN1-mitochondrial phenotypes (Figure 3B and C; eFigure 1 in the Supplement).^21^ Mitochondrial size and intensity were defective to the same degree in cells expressing p.Ala20Ser and p.Cys133Trp. These defects were reversed to the wild-type phenotype on serine supplementation in the culture (Figure 3B and C; eFigure 1 in the Supplement).

### *SPTLC1* Variants in Patients With Adult-Onset ALS

Having established that variants in *SPTLC1* are associated with juvenile ALS, we explored the role of variation in this gene in the pathogenesis of adult-onset ALS by evaluating the occurrence of *SPTLC1* variants in a series of 6258 patients with adult-onset ALS. This screening identified 20 novel *SPTLC1* variants in 23 patients with ALS (0.4%) that were rare or absent in healthy controls and were predicted to be damaging (eTable 3 and eFigures 2 and 3 in the Supplement). The typical clinical features of ALS were observed among these patients with adult-onset disease, and none of the patients reported sensory or autonomic involvement (eTable 4 in the Supplement). The intensity and number of motor neurons staining with SPTLC1 were diminished in autopsy tissue obtained from a patient with ALS carrying a p.Arg445Gln variant in *SPTLC1* (eFigure 4 in the Supplement). Gene burden testing was not significant for *SPTLC1* variants as a cause of adult-onset ALS (87 variants in population samples; uncorrected 1-sided Fisher test *P* value using TRAPD software package = 1.9 × 10^−4^; not significant after correction for multiple testing of 20 000 genes).

## Discussion

We provide genetic, biochemical, and cellular data that variations in *SPTLC1* are associated with juvenile ALS. First, we found 3 unrelated patients diagnosed with juvenile ALS who carried de novo variants in *SPTLC1* and identified a fourth patient with juvenile ALS carrying another *SPTLC1* variant for whom inheritance could not be determined. These variants were not present in our in-house control data set or in online databases of human variants, indicating they were rare variants in diverse populations. Two of the patients carried the same alanine to serine amino acid shift at position 20 of the protein, which arose from different nucleotide changes. Second, cell-based assays of SPT activity confirmed that the p.Ala20Ser variation altered the encoded enzyme’s function, leading to increased aberrant utilization of alanine and glycine as substrates. This biochemical pattern was consistent with a mechanism reported in patients with HSAN1 caused by *SPTLC1* variants.^20^ Third, we used immunohistochemistry to demonstrate that SPTLC1 is abundantly expressed within the motor neurons of healthy spinal cord tissue.

Though labeled as HSAN1, the phenotypes associated with variants in *SPTLC1* are varied, with patients manifesting various combinations of sensory loss, autonomic dysfunction, and motor weakness.^22^ Indeed, there is a previous report of a de novo p.Ser331Tyr variant in *SPTLC1* in a young French girl presenting with a similar phenotype to the patients in this article.^16^ Her clinical picture consisted of severe growth restriction, cognitive impairment, amyotrophy, hyperreflexia, vocal cord paralysis, and respiratory failure, although this patient was not diagnosed as having juvenile ALS. More recently, retinal disease has been reported in patients carrying *SPTLC1* variants.^23^ This clinical heterogeneity has been linked to the differing effects of each variant on SPTLC1 enzyme-substrate preference,^20^ and we observed similar differences in substrate utilization across the variants that we had studied at the enzymatic level (Figure 3A). Alternatively, the phenotypes associated with variants in HSAN1 may represent a continuum between sensory neuropathy and ALS. Future postmortem studies that determine the central nervous system pathology (eg, TAR DNA-binding protein 43, tau, β-amyloid deposition) underlying the motor neuron deficits and the cognitive impairment may resolve the nature of this overlap with other neurodegenerative diseases.

Perturbed sphingolipid metabolism underlies many neurological disorders, such as Niemann-Pick disease and Gaucher disease,^24^ and may play a role in the pathogenesis of Alzheimer disease.^25^ Sphingolipid metabolism has also been implicated in motor neuron degeneration. For example, patients with partial deficiency of hexosaminidase A enzyme activity (also known as GM2 gangliosidosis, a form of sphingolipidosis) may have clinical manifestations mimicking ALS.^26^ The accumulation of ceramides and cholesterol esters also occurs within the spinal cords of patients with ALS and an *SOD1* transgenic mouse model of ALS.^27^

Owing to the poor prognosis observed among patients with juvenile ALS and work published by other groups,^20,28^ patient 2 was commenced on high-dose (10 g per day) oral serine supplementation on a compassionate basis. Her body weight increased during this off-label treatment, which was the first time she had gained weight in several years. The patient’s ceramide levels were within normal range and trending downwards, indicating that ceramide toxic effects, a theoretical possibility with serine treatment, were not present (eFigure 5 and eTable 5 in the Supplement). We did not observe evidence of neurological improvement, although prolonged therapy would be required to detect such an effect.^29^

Serine is a nonessential amino acid that is available as a low-cost nutritional supplement. A 10% serine-enriched diet was associated with a reduction in neurotoxic deoxysphingolipid plasma levels both in transgenic mice expressing the p.Cys133Trp *SPTLC1* variant and in human patients diagnosed with HSAN1.^28^ Furthermore, a safety trial involving 20 patients with adult-onset ALS demonstrated that high doses of oral serine are well tolerated and that this polar amino acid is actively transported across the blood-brain barrier.^30^ Nutritional supplementation has proven to be remarkably effective in other forms of ALS.^31^ Despite these supportive data, future clinical trials are needed to determine the effectiveness and safety profile of serine supplementation in patients with juvenile ALS owing to *SPTLC1* variants.

### Limitations

Our study had limitations. DNA was not available from the parents of patient 4, so it was not possible to determine whether or not the variation arose spontaneously. Nevertheless, the lack of a family history supports the possibility that this variant was de novo in origin; there is only a 3.1% chance that none of her 5 siblings would have inherited an autosomal-dominant variant from a transmitting parent. Our evidence also demonstrates that variants in *SPTLC1* are not a common cause of adult-onset ALS. Overall, our data imply that the genetic causes of juvenile ALS and adult-onset ALS are distinct.

## Conclusions

In conclusion, our data broaden the phenotype associated with variants in *SPTLC1* to include juvenile ALS and implicate sphingolipid metabolism as a pathway in motor neuron disease. Our findings are relevant in light of the fact that nutritional supplementation with serine has been postulated to ameliorate the toxic effect of abnormal sphingolipid metabolites if instituted at an early stage in the disease.^28^ In such cases, abnormal plasma metabolites could be used as a marker of target engagement.^32^ This provides an early opportunity for future clinical trials to test the precision medicine approach in an otherwise fatal neurodegenerative disease.

## Notes

Supplement.
eMethods.
**eFigure 1. **Assays of SPT activity and mitochondrial cellular phenotypes in cell models. **eFigure 2. ***SPTLC1* mutations detected in adult-onset ALS patients. **eFigure 3. **Chromatograms of *SPTLC1* variants identified in patients. **eFigure 4. **Immunohistochemistry of SPTLC1 in the lumbar spinal cord. **eFigure 5. **Time-series changes of complex sphingolipids in patient 2 on high-dose oral serine supplementation. **eTable 1. **Demographic and clinical features of adult-onset ALS cases screened for *SPTLC1* mutations. **eTable 2. **Primer sequences used for Sanger sequencing and mutagenesis of *SPTLC1*. **eTable 3. ***SPTLC1* mutations identified in adult-onset ALS cases. **eTable 4. **Demographic and clinical features of adult-onset ALS cases carrying *SPTLC1* mutations. **eTable 5. **Complex sphingolipid plasma levels in ALS cases and controls.
eReferences.
