# Supplementary material for: Association of Variants in the SPTLC1 Gene With Juvenile Amyotrophic Lateral Sclerosis
Source: JAMA Neurol. 2021 Aug 30;78(10):1–13. doi: 10.1001/jamaneurol.2021.2598 (PMC8406220; doi:10.1001/jamaneurol.2021.2598)
Supplement: Supplement. — eMethods. eFigure 1. Assays of SPT activity and mitochondrial cellular phenotypes in cell models. eFigure 2. SPTLC1 mutations detected in adult-onset ALS patients. eFigure 3. Chromatograms of SPTLC1 variants identified in patients. eFigure 4. Immunohistochemistry of SPTLC1 in the lumbar spinal cord. eFigure 5. Time-series changes of complex sphingolipids in patient 2 on high-dose oral serine supplementation. eTable 1. Demographic and clinical features of adult-onset ALS cases screened for SPTLC1 mutations. eTable 2. Primer sequences used for Sanger sequencing and mutagenesis of SPTLC1. eTable 3. SPTLC1 mutations identified in adult-onset ALS cases. eTable 4. Demographic and clinical features of adult-onset ALS cases carrying SPTLC1 mutations. eTable 5. Complex sphingolipid plasma levels in ALS cases and controls. eReferences. [file jamaneurol-e212598-s001.pdf]

# Supplemental Online Content

Johnson JO, Chia R, Miller DE, et al; FALS Sequencing Consortium; American Genome Center; International ALS Genomics Consortium; ITALSGEN Consortium. Association of variants in the *SPTLC1* gene with juvenile amyotrophic lateral sclerosis. *JAMA Neurol*. Published online August 30, 2021. doi:10.1001/jamaneurol.2021.2598

**eMethods.**
**eAppendix.** Consortia authors and affiliations
**eFigure 1.** Assays of SPT activity and mitochondrial cellular phenotypes in cell models
**eFigure 2.** *SPTLC1* mutations detected in adult-onset ALS patients
**eFigure 3.** Chromatograms of *SPTLC1* variants identified in patients
**eFigure 4.** Immunohistochemistry of SPTLC1 in the lumbar spinal cord
**eFigure 5.** Time-series changes of complex sphingolipids in patient 2 on high-dose oral serine supplementation
**eTable 1.** Demographic and clinical features of adult-onset ALS cases screened for *SPTLC1* mutations
**eTable 2.** Primer sequences used for Sanger sequencing and mutagenesis of *SPTLC1*
**eTable 3.** *SPTLC1* mutations identified in adult-onset ALS cases
**eTable 4.** Demographic and clinical features of adult-onset ALS cases carrying *SPTLC1* mutations
**eTable 5.** Complex sphingolipid plasma levels in ALS cases and controls
**eReferences.**

This supplementary material has been provided by the authors to give readers additional information about their work.

© 2021 Johnson JO et al. *JAMA Neurology*.

## eMethods.

**Detailed description of patient 1**

Patient 1 is a right-handed, white woman who had presented at five years of age with spastic diplegia and motor delay. There was no family history of neurological disease, and her three siblings and her parents were neurologically healthy (see **Figure 2A**). By age 8, she had developed tongue fasciculations and proximal limb weakness. Her weight and height were consistently below the $10^{th}$ percentile despite treatment with growth hormones and oxandrolone. A gastrostomy tube was placed at age 10 due to anorexia.

Her muscle weakness progressed over the years. By age 19, she could not sit unsupported, stand or walk, and was entirely dependent for activities of daily living. Her hand weakness had progressed to the point that she could no longer write or use an iPad, and she had increasing difficulty using the joystick to control her motorized wheelchair. She complained of frequent and severe nocturnal leg spasms requiring treatment with valium, gabapentin, and trazodone.

She underwent tracheostomy at 17 years of age, and she has been on continuous ventilatory support since that time. The same year, she underwent spinal fusion surgery to correct scoliosis and lordosis that was causing restrictive lung disease, and right hip surgery for contractures. At age 18, she developed nighttime urinary urgency and intermittent bradycardia, likely secondary to increased vagal tone.

At age 16, the patient was referred to psychiatry to evaluate depression, anxiety, and insomnia. At age 18, her mother noted that the patient was not as "witty and bright" as she had been. At age 20, the patient was having difficulty remembering things and was slow to speak in social settings.

Neurophysiology testing at ages 9, 10, and again at age 13 revealed diffuse chronic denervation, consistent with anterior horn cell dysfunction. Right sural nerve conduction amplitude and velocity were consistently reported to be normal. Cool detection threshold (CDT) and Q-Sweat testing, a commercial quantitative sweat measurement system, did not show evidence of small fiber sensory or autonomic dysfunction in distal peripheral nerves at the age of 20. Muscle biopsy of the left quadriceps at age 11 revealed chronic denervation and reinnervation with extensive atrophy involving entire fascicles and scattered large type 1 fibers.

Examination at the age of 20 revealed a short woman (122 cm, weight 28 kg, body mass index 18.8 kg/m$^2$, z-score = -1.12, $13^{th}$ percentile) sitting in a motorized wheelchair and ventilated via tracheostomy. The patient was dyspneic on talking, and she was moderately dysarthric. Her affect was jocular.

Montreal Cognitive Assessment yielded a score of 11 out of 25 due to poor orientation to date and location, poor serial sevens, impaired generation of words beginning with "F", poor abstraction, and poor delayed recall. Other visuospatial and executive functions were not assessed due to the patient's inability to write. Frontal Behavioral Inventory completed by the patient's mother gave a score of 7 [normal range: 0-27]. Frontotemporal Dementia Rating Scale, also completed by the patient's mother, identified a loss of interest, impulsivity, forgetfulness, and urinary and fecal incontinence (likely neurological in etiology, rather than arising from a behavioral issue).

Cranial nerve examination showed decreased upward eye gaze, bilateral fine nystagmus on lateral gaze, prominent perioral fasciculations, and a slow, wasted, fasciculating tongue. Palatal movements were also slow, and jaw jerk was absent. Shoulder shrug, neck flexion, and neck extension were weak (MRC = 3/5).

There was severe muscle wasting and weakness involving all four limbs. She had contractures of both feet with flexion of toes and foot inversion. Fasciculations were not observed. Ankle reflexes were brisk bilaterally, whereas knee jerks and upper limb reflexes were absent. Plantar responses were mute on Babinski testing (toe dorsiflexion and plantar flexion were 0/5). Pain sensation was decreased in the right arm and left leg. Temperature sensation, proprioception, and vibration sensation were normal.

© 2021 Johnson JO et al. *JAMA Neurology*.

**Detailed description of patient 2**

Patient 2 was a right-handed teenage girl with mixed African American and white race/ethnicity who had developed difficulty walking due to lower limb muscle weakness before the age of 10. Over the next few years, she had trouble climbing stairs, and tongue fasciculations were observed. She had generalized weakness and muscle wasting at the time of her referral in her mid-teens. She could not walk long distances without fatigue, and her weight was more than 7 standard deviations below average for her age. A prior endocrinology workup for her failure to gain weight was unremarkable. Her school performance had also begun to decline in her mid-teens.

Past medical history was otherwise unremarkable, and there was no family history of neurodegenerative disorders (**Figure 2B**).

Examination at presentation revealed a thin girl with a body mass index below the 1$^{st}$ percentile based on her age and gender (z-score = -7). There was no evidence of orthostatic hypotension. Cranial nerve examination revealed tongue fasciculations and wasting (**Figure 1A**).

Limb examination showed an exaggerated lumbar lordosis, generalized weakness involving all muscles, and prominent scapula winging bilaterally (**Figure 1B**). Her upper limb reflexes were diminished. In the lower limbs, knee jerks were reduced, and ankle jerks were asymmetrically brisk. Pinprick, temperature, vibration, and proprioception sensations were normal. The patient had an abnormal gait and a floridly positive Gower's sign.

MRI imaging of the brain and spine were unremarkable, though these imaging studies showed diffusely diminished pelvic and thigh musculature and a notable lack of subcutaneous fat. EMG revealed active and chronic denervation with prominent complex repetitive discharges and giant muscle unit action potentials consistent with a long-standing motor neuropathic or neuronopathic disorder. There was no neurophysiological evidence for a sensory neuropathy. Plasma metanephrine and normetanephrine were normal. A neuropsychological evaluation revealed that her intellectual functioning was within the average range based on her age. However, she demonstrated significant challenges with sustained attention and stamina, aspects of executive functioning, and motor coordination. A swallow study was unremarkable.

**Detailed description of patient 3**

Patient 3 was an eleven-year-old African American right-handed girl with a history of failure to gain weight and toe-walking since the age of four. She was first evaluated at the age of 10 because of difficulty walking due to her left foot "turning in." In retrospect, her gait had been gradually deteriorating over several years, resulting in occasional falls and difficulty walking upstairs. She was no longer able to participate in her school dance class due to weakness and exercise-induced cramps. Subsequently, her symptoms had spread to her hands. She was unable to undo soda bottle tops, and dressing has been taking longer than usual. The patient had complained of tingling in her feet over the last three years, but the numbness was only noticed on her first neurological evaluation. She had a painless ulcer on her right foot that developed from her ankle-foot orthosis. She has occasional choking episodes, mostly on liquids. She has noticed the pooling of secretions. Her speech has been normal, and there have been no obvious cognitive or behavioral problems. She has worn glasses since age eight. Ophthalmological examination at age eleven revealed bilateral cataracts that did not require surgical intervention. The patient has a history of profuse sweating, but no urinary symptoms or dizziness on standing.

Her past medical history included adenoidectomy, bilateral Achilles tenotomy at the age of 10, and long-standing vitamin D deficiency requiring oral supplementation. She was born one month premature, but she achieved her early developmental milestones appropriately. There was no family history of ALS, and her nineteen-year-old brother was healthy (**Figure 2C**). Her maternal grandmother, paternal grandfather, and maternal aunt were diagnosed with late-onset dementia.

Examination at the age of eleven revealed a thin girl with a body mass index of 10.9 kg/m2, representing less than the 1$^{st}$ percentile based on her age and gender (z score = -6.5). Blood pressure was 111/62 mmHg with a heart rate of 107 beats per minute while lying, and 128/62 mmHg with a heart rate of 139 beats per minute on standing. She was alert and articulate, and her conversational speech was normal, though she had difficulty forming K and L sounds.

© 2021 Johnson JO et al. *JAMA Neurology*.

On cranial nerve examination, her near visual acuity was 20/50 bilaterally without glasses, and fundoscopy confirmed the presence of lens opacities. The tongue was wasted, weak, and slow to move with prominent fasciculations (**Figure 1C-D**). Jaw jerk was present, and shoulder shrug was weak bilaterally. The rest of the cranial nerves were intact.

Her posture was normal, and there was no evidence of scoliosis. The examination of her limbs showed generalized atrophy and hypotonia. Fasciculations were present in the upper and lower limbs. There was a left greater than right pronator drift with bilateral postural tremor. The patient had bilateral hammertoes, high arches, and a healing ulcer over the right first metatarsal joint. Strength was decreased in a pyramidal pattern that was asymmetric and affecting distal muscles more than proximal. Power in the upper limbs was as follows: bilateral shoulder abductors = 4/5, shoulder adductors = 5/5, right elbow flexor = 5/5, left elbow flexor = 4/5, bilateral elbow extensors = 4/5, wrist dorsiflexor = 4/5, wrist palmar flexion = 5/5, finger flexors = 4/5, right finger extensors = 4/5, left finger extensors = 3/5, bilateral finger adductors = 2/5, finger abductors = 3/5, and thumb abductors = 4/5. Power in the lower limbs was as follows: bilateral hip flexors = 4/5, hip abductors = 4/5, hip adductors = 5/5, knee flexors = 4/5, knee extensors = 5/5, ankle dorsiflexion = 3/5, ankle plantar flexion = 5/5, ankle eversion = 3/5, ankle inversion = 5/5, hallux flexors = 2/5, and hallux extensors = 2/5. Reflexes in the upper limbs were 4+ with spread with a positive Hoffman's sign bilaterally. In the lower limbs, knee jerks were 3+ bilaterally, and ankle jerks were absent. The left Babinski was down going, whereas the right Babinski elicited fanning of her toes. The patient was ataxic on heel-to-shin and finger-to-nose testing with bilateral dysdiadochokinesis, though this appeared to be secondary to muscle weakness. The sensory exam revealed decreased pinprick and temperature sensation in the upper limbs to the level of the wrist, right worse than left, and in the lower limbs to the mid-calf level. Proprioception and vibration sensations were intact. The patient walked abnormally due to generalized weakness and bilateral foot drop. She had difficulty with tandem gait testing, was able to stand on her toes, but she was unable to stand on her heels. Romberg's sign was negative. She had a positive Gower's sign.

Neurophysiological examination at the age of 11 was consistent with sensorimotor axonal neuropathy. Electromyography of the left vastus lateralis and tibialis anterior muscles showed large amplitude, polyphasic potentials with a reduced amplitude pattern. MRI of the brain and spinal cord was unremarkable. Pulmonary function testing demonstrated a mild restrictive ventilatory defect (forced expiratory volume in one second-to-forced vital capacity ratio = 0.9). Creatine phosphokinase, vitamin B12, and folate serum transferrin, iron, copper, metabolic screens were within normal limits. Serine level was within the normal range at 79.3 umol/l.

**Patient 4**

Patient 4 was a thirty-four-year-old, right-handed, Turkish woman with a history of arm and leg weakness and atrophy since the age of fifteen. Her five siblings, parents, paternal and maternal grandparents, and extended family did not report neurological symptoms. Her mother died at the age of 65, and her father died at the age of 50.

**Recruitment**

Patients 1, 2, and 3 were followed by their local neurologists and geneticists. All patients were diagnosed with juvenile ALS by their local physicians. Patients 1 (and her family) was referred to Dr. Traynor at the NIH Clinical Center for evaluation by her pediatric neurologist. Dr. Traynor was contacted by the physicians of patients 2 and 3 through the GeneMatcher program at GeneDx.[1] Patient 2 was followed at her local hospital. Patient 3 (and her family) was referred to Dr. Traynor at the NIH Clinical Center for evaluation by her pediatrician.

Patients 1 and 3 are the only juvenile ALS cases that Dr. Traynor has seen in his ALS clinic since moving to the NIH in 2005. In a separate project, deidentified DNA from three other trios where the proband had juvenile ALS of unknown genetic etiology was sent to Dr. Traynor's laboratory and underwent exome sequencing. The ages of symptom onset in these samples were older than those carrying *SPTLC1* mutations (age of onset was 20 years for Coriell repository sample ND14489, 22 years of age for the Italian sample SLA2010-483, and 24 years of age for Italian sample SLA2011-110). De novo *SPTLC1* mutations were not identified in these trio samples.

Patient 4 was identified by Dr. Başak working at the Koç University, Istanbul, Turkey in a collection of 52 individuals diagnosed with juvenile ALS undergoing whole-exome sequencing.

**Immunohistochemistry**

The control spinal cord was obtained from the postmortem of a 64-year-old white man, and the postmortem interval was 28 hours. The sporadic ALS spinal cord was obtained from the postmortem of a 66-year old white male, and the postmortem interval was 4.5 hours. The spinal cord carrying the *SPTLC1* p.Arg445Gln mutation was obtained from the postmortem of a 78-year-old white man who had been diagnosed with sporadic ALS. The postmortem interval was 3.83 hours, and survival from symptom onset in his upper limbs was 68 months. The autopsy of the *SPTLC1* carrier was performed according to the research protocol of the Department of Veterans Affairs Biorepository Brain Bank.[2] The other autopsies were performed at the JHMI Brain Resource Center according to their internal protocol. Formalin-fixed, paraffin-embedded blocks were sectioned to 5 um thickness and stained with primary anti-SPTLC1 antibody (Sigma-Aldrich) at 1:50 dilution overnight at 4°C and counter-stained with hematoxylin.

**Immunopurification of SPTLC1 protein complex**

SPTLC1-FLAG proteins were immuno-purified from HEK293FT cells that were stably expressing SPTLC1. Cells were lysed in lysis buffer (50 mM HEPES, pH8.0, 1 mM EDTA, 0.1% (w/v) sodium monolaurate, phosphatase (Thermo Fisher), and protease inhibitor (Roche AG)). Membrane proteins were further solubilized by sonication for fifteen seconds at 50% power and 50% pulsation for a total of 45 seconds. The lysate was clarified by centrifugation at 21,000g for five minutes at 4°C to remove large cellular debris, followed by immunoprecipitation with EZview Red Anti-Flag M2 Affinity Gel (Sigma-Aldrich) as previously described.[3] Purified SPTLC1-FLAG was quantified against a bovine serum albumin protein standard curve (BSA, Thermo Fisher) ran on an SDS-PAGE gel and transferred to a nitrocellulose membrane (Bio-rad). The membrane was stained with Revert Total Protein Stain (LI-COR) and imaged on an Odyssey CLx imaging system. Interaction of SPTLC2 with SPTLC1-FLAG was confirmed by Western blot showing that the purified serine palmitoyltransferase complex was likely functional.

**Photometric serine palmitoyltransferase enzymatic assay**

Condensation of serine and palmitoyl-CoA by serine palmitoyltransferase enzyme produces 3-ketodihydrosphingosine and releases both carbon dioxide and free coenzyme A (CoA). This photometric assay measures the amount of free CoA released that is reactive to 5,5'-dithiobis-2-nitrobenzoic acid (DTNB, Thermo Fisher), which reflects the enzymatic activity of serine palmitoyltransferase.

This assay was adapted from and shown to be comparable to radioactive assays measuring radiolabeled 3-ketodihydrosphingosine.[4] Triplicate wells were assayed per 100ng of purified wild-type or mutant SPTLC1 proteins, and per amino acid tested. The reaction product was measured at 412 nm at the zero-time point and every two minutes for up to one hour. In place of purified SPTLC1-FLAG protein, varying concentrations of CoA were included to build the calibration curve for the estimation of CoA released from the serine palmitoyltransferase enzymatic reaction. Estimated amounts of CoA produced per nanogram of SPTLC1-FLAG protein were plotted over time for each amino acid.

**Western blotting**

Western blot was performed as previously described.[3] We used the following primary antibodies at the indicated dilutions: mouse anti-Flag (1:5,000, Sigma-Aldrich), rabbit anti-SPTLC1 (1:1,000, Sigma-Aldrich), and rabbit anti-SPTLC2 (1:1000, LifeSpan BioSciences Inc.).

© 2021 Johnson JO et al. *JAMA Neurology*.

**Data and materials availability**

Alzheimer's Disease Sequencing Project (ADSP): https://www.niagads.org/adsp/content/home. Database of Genotypes and Phenotypes (dbGaP): https://www.ncbi.nlm.nih.gov/gap. Genome Aggregation Database (gnomAD): https://gnomad.broadinstitute.org. Haplotype Reference Consortium (HRC): www.haplotype-reference-consortium.org. Kaviar Genomic Variant Database: http://db.systemsbiology.net/kaviar/. ANNOVAR: annovar.openbioinformatics.org. Clustal Omega: www.ebi.ac.uk/Tools/msa/clustalo/. GATK: http://www.broadinstitute.org/gsa/wiki/index.php/Home_Page. MassLynx: http://www.waters.com/waters/en_US/MassLynx-Mass-Spectrometry-Software-/nav.htm?cid=513164&locale=en_US. Picard: http://broadinstitute.github.io/picard. PLINK: https://www.cog-genomics.org/plink/1.9/. Sequencher: http://genecodes.com. TRAPD: https://github.com/mhguo1/TRAPD.

© 2021 Johnson JO et al. *JAMA Neurology*.

**eAppendix.** Consortia authors and affiliations

**The members of the FALS Sequencing Consortium are:**

Bradley N. Smith, PhD[1], Nicola Ticozzi, MD, PhD[2,3], Claudia Fallini, PhD[4], Athina Soragia Gkazi, PhD[1], Simon D. Topp, MSc[1,5], Emma L. Scotter, PhD[6], Kevin P. Kenna, PhD[4], Pamela Keagle, BS[4], Cinzia Tiloca, PhD[7], Caroline Vance, PhD[8], Claire Troakes, PhD[8], Claudia Colombrita[7], Andrew King, PhD[1], Viviana Pensato, PhD[9], Barbara Castellotti, PhD[9], Frank Baas, MD, PhD[10], Anneloor L.M.A. ten Asbroek, PhD[11], Diane McKenna-Yasek, RN[4], Russell L. McLaughlin, PhD[12], Meraida Polak, RN[13], Seneshaw Asress, MSc[13], Jesús Esteban-Pérez, MD[14], Zorica Stevic, MD, PhD[15], Sandra D'Alfonso, PhD[16], Letizia Mazzini, MD[17], Giacomo P. Comi, MD[18], Roberto Del Bo, PhD[18], Mauro Ceroni, MD[19], Stella Gagliardi, PhD[20], Giorgia Querin, MD[21], Cinzia Bertolin, PhD[21], Wouter van Rheenen, MD[22], Rosa Rademakers, PhD[23], Marka van Blitterswijk, MD, PhD[23], Giuseppe Lauria, MD[24], Stefano Duga, PhD[25], Stefania Corti, MD, PhD[18], Cristina Cereda, PhD[20], Lucia Corrado, PhD[16], Gianni Sorarù, MD, PhD[21], Kelly L. Williams, PhD[26], Garth A. Nicholson, PhD[26], Ian P. Blair, PhD[26], Claire Leblond-Manry, PhD[27], Guy A. Rouleau, MD, PhD[28], Orla Hardiman, MD[29], Karen E. Morrison, MD[30], Jan H. Veldink, MD, PhD[22], Leonard H. van den Berg, MD, PhD[22], Ammar Al-Chalabi, PhD, FRCP[8], Hardev Pall, MD[31], Pamela J. Shaw, MD[32], Martin R. Turner, MD[33], Kevin Talbot, MD[33], Franco Taroni, MD[9], Alberto García-Redondo, PhD[14], Zheyang Wu, PhD[34], Jonathan D. Glass, MD[13], Cinzia Gellera, PhD[9], Antonia Ratti, PhD[2], Robert H. Brown, Jr., MD, Dphil[4], Vincenzo Silani, MD[2,3], Christopher E. Shaw, MD[8,5], John E. Landers, PhD[4]

1. Centre for Neurodegeneration Research, King's College London, Department of Clinical Neuroscience, Institute of Psychiatry, London, UK.
2. Department of Neurology and Laboratory of Neuroscience, IRCCS Istituto Auxologico Italiano, Milan 20149, Italy.
3. Department of Pathophysiology and Transplantation, "Dino Ferrari" Center, Università degli Studi di Milano, Milan 20122, Italy.
4. Department of Neurology, University of Massachusetts Medical School, Worcester, MA 01605, USA.
5. UK Dementia Research Institute at King's College London, London, UK.
6. Centre for Brain Research, University of Auckland, New Zealand.
7. Department of Neurology, IRCCS Istituto Auxologico Italiano, Milan, Italy.
8. Maurice Wohl Clinical Neuroscience Institute, Department of Basic and Clinical Neuroscience, King's College London, London SE5 9RS, UK.
9. Unit of Medical Genetics and Neurogenetics, Fondazione IRCCS Istituto Neurologico 'Carlo Besta', Milan 20133, Italy.
10. Clinical Genetics, Leiden University Medical Center, Leiden, the Netherlands.
11. Department of Neurogenetics and Neurology, Academic Medical Centre, Amsterdam, the Netherlands.
12. Population Genetics Laboratory, Smurfit Institute of Genetics, Trinity College Dublin, Dublin, Republic of Ireland.
13. Department of Neurology, Emory University, Atlanta, GA 30322, USA.
14. Unidad de ELA, Instituto de Investigación Hospital 12 de Octubre de Madrid, SERMAS, and Centro de Investigación Biomédica en Red de Enfermedades Raras (CIBERER U-723), Madrid, Spain.
15. Neurology Clinic, Clinical Center of Serbia School of Medicine, University of Belgrade, Serbia.
16. Department of Health Sciences, University of Eastern Piedmont, Novara, Italy.
17. ALS Center, Azienda Ospedaliero Universitaria Maggiore della Carità, Novara, Italy.
18. Neurology Unit, IRCCS Foundation Ca' Granda Ospedale Maggiore Policlinico, Milan, Italy.
19. Department of Brain and Behavior, University of Pavia, 27100 Pavia, Italy and General Neurology Unit, IRCCS Mondino Foundation, 27100 Pavia, Italy.
20. Genomic and post-Genomic Center, Mondino Foundation – IRCCS, 2 - 27100 Pavia.
21. Department of Neurosciences, University of Padova, Padova, Italy.
22. Department of Neurology, Brain Center Rudolf Magnus, University Medical Center Utrecht, Utrecht, the Netherlands.
23. Department of Neuroscience, Mayo Clinic, Jacksonville, FL 32224, USA.
24. 3rd Neurology Unit, Motor Neuron Diseases Center, Fondazione IRCCS Istituto Neurologico 'Carlo Besta', Milan, Italy.
25. Humanitas Clinical and Research Center – IRCCS (Mi) Italy and Humanitas University, Department of Biomedical Sciences, Milan, Italy.

© 2021 Johnson JO et al. *JAMA Neurology*.

26. Centre for Motor Neuron Disease Research, Department of Biomedical Sciences, Faculty of Medicine and Health Sciences, Macquarie University, Sydney, New South Wales, Australia.
27. Human Genetics and Cognitive Functions Unit, Institut Pasteur, Paris, France.
28. Montreal Neurological Institute, Department of Neurology and Neurosurgery, McGill University, Montreal, Quebec, Canada.
29. Academic Unit of Neurology, Trinity Biomedical Sciences Institute, Trinity College Dublin, Dublin, Republic of Ireland.
30. Faculty of Medicine, Health and Life Sciences, Queen's University Belfast, UK.
31. School of Clinical and Experimental Medicine, University of Birmingham, Birmingham, UK.
32. Sheffield Institute for Translational Neuroscience, Department of Neuroscience, University of Sheffield, Sheffield S10 2HQ, UK.
33. Nuffield Department of Clinical Neurosciences, University of Oxford, UK.
34. Department of Bioinformatics and Computational Biology, Worcester Polytechnic Institute, Worcester, MA 01609, USA.

**The members of The American Genome Center are:**

Clifton L. Dalgard, PhD[1,2], Adelani Adeleye[1,3], Anthony R. Soltis, PhD[1,3], Camille Alba, MS[1,3], Coralie Viollet, PhD[1,3], Dagmar Bacikova[1,3], Daniel N. Hupalo, PhD[1,3], Gauthaman Sukumar, MS[1,3], Harvey B. Pollard, MD, PhD[1,2], Matthew D. Wilkerson, PhD[1,2,3], Elisa McGrath Martinez[1,3]

1. The American Genome Center, Collaborative Health Initiative Research Program, Uniformed Services University of the Health Sciences, Bethesda, MD 20814, USA
2. Department of Anatomy, Physiology & Genetics, Uniformed Services University of the Health Sciences, Bethesda, MD 20814, USA
3. Henry M. Jackson Foundation for the Advancement of Military Medicine, Bethesda, MD, 20817

**The members of the international ALS genomics consortium (iALSgc) are:**

Yevgeniya Abramzon, BS[1,2], Sarah Ahmed, BS[3], Sampath Arepalli, MS[4], Robert H. Baloh, MD[5], Robert Bowser, PhD[6], Christopher B. Brady, PhD[7], Alexis Brice, MD[8,9], James Broach, PhD[10], Roy H. Campbell, PhD[11], William Camu, MD[12], Ruth Chia, PhD[1], John Cooper-Knock, MD[13], Jinhui Ding, PhD[14], Carsten Drepper, PhD[15], Vivian E. Drory, MD[16], Travis L. Dunckley, PhD[17], John D. Eicher, PhD[18], Bryce K. England, BS[19], Faraz Faghri, BS[20,11], Eva Feldman, MD[21], Mary Kay Floeter, MD[22], Pietro Fratta, MD[2], Joshua T. Geiger, MD[3], Glenn Gerhard, MD[23], J. Raphael Gibbs, PhD[14], Summer B. Gibson, MD[24], Jonathan D. Glass, MD[25], John Hardy, PhD[26], Matthew B. Harms, MD[27], Terry D. Heiman-Patterson, MD[28,29], Dena G. Hernandez, PhD[4], Lilja Jansson[30], Janine Kirby, PhD[13], Neil W. Kowall, MD[31], Hannu Laaksovirta, MD[30], Natalie Landeck, PhD[32], Francesco Landi, MD[33], Isabelle Le Ber, MD, PhD[8,9], Serge Lumbroso, MD[34], Daniel JL. MacGowan, MD[35], Nicholas J. Maragakis, MD[36], Gabriele Mora, MD[37], Kevin Mouzat, MD[34], Natalie A. Murphy, BS[1], Liisa Myllykangas, MD, PhD[38], Mike A. Nalls, PhD[20,39], Richard W. Orrell, MD[40], Lyle W. Ostrow, MD[36], Roger Pamphlett, MD[41], Stuart Pickering-Brown, PhD[42], Erik P. Pioro, MD[43], Olga Pletnikova, MD[44], Hannah A. Pliner, PhD[1], Stefan M. Pulst, MD[24], John M. Ravits, MD[45], Alan E. Renton, PhD[1,46], Alberto Rivera, BS[1], Wim Robberecht, MD[47], Ekaterina Rogaeva, PhD[48], Sara Rollinson, PhD[42], Jeffrey D. Rothstein, MD, PhD[36], Sonja W. Scholz, MD, PhD[3,36], Michael Sendtner, MD[49], Pamela J. Shaw, MD[13], Katie C. Sidle, MD[26], Zachary Simmons, MD[50], Andrew B. Singleton, PhD[20], Nathan Smith, PhD[32], David J. Stone, PhD[18], Pentti J. Tienari, MD, PhD[30], Juan C. Troncoso, MD[44], Miko Valori, PhD[30], Philip Van Damme, MD[47,51], Vivianna M. Van Deerlin, MD, PhD[52], Ludo Van Den Bosch, PhD[47], Lorne Zinman, MD[53], John E. Landers, PhD[54], Adriano Chiò, MD[55,56], Bryan J. Traynor, MD, PhD[1,26,36,57]

1. Neuromuscular Diseases Research Section, Laboratory of Neurogenetics, National Institute on Aging, Bethesda, MD 20892, USA

© 2021 Johnson JO et al. *JAMA Neurology*.

2. Sobell Department of Motor Neuroscience and Movement Disorders, Institute of Neurology, University College London, London, WC1N 3BG, UK
3. Neurodegenerative Diseases Research Unit, Laboratory of Neurogenetics, National Institute of Neurological Disorders and Stroke, Bethesda, MD 20892, USA
4. Genomics Technology Group, Laboratory of Neurogenetics, National Institute on Aging, Bethesda, MD 20892, USA
5. Department of Neurology, Cedars-Sinai Medical Center, Los Angeles, CA 90048, USA
6. Division of Neurology, Barrow Neurological Institute, Phoenix, AZ 85013, USA
7. Research and Development Service, Veterans Affairs Boston Healthcare System, Boston, MA 02130, USA
8. Centre de Recherche de l'Institut du Cerveau et de la Moelle épinière, Université Pierre et Marie Curie, Paris, France
9. INSERM U975, Paris, France
10. Department of Biochemistry, Penn State College of Medicine, Hershey, PA 17033, USA
11. Department of Computer Science, University of Illinois at Urbana-Champaign, 201 North Goodwin Avenue, Urbana, IL 61801, USA
12. ALS Reference Center, Gui de Chauliac Hospital, CHU and Univ Montpellier, Montpellier France, Montpellier, France
13. Department of Neuroscience, University of Sheffield, Sheffield, S10 2HQ, UK
14. Computational Biology Group, Laboratory of Neurogenetics, National Institute on Aging, Bethesda, MD 20892, USA
15. Institute for Clinical Neurobiology, University of Würzburg, Würzburg, D-97078, Germany
16. Department of Neurology, Tel-Aviv Sourasky Medical Center, Tel-Aviv, Israel
17. Neurodegenerative Disease Research Center, Biodesign Institute, Arizona State University, 1001 S. McAllister Ave, Tempe, AZ 85281, USA
18. Genetics, Genetics and Pharmacogenomics, Merck Research Laboratories, Merck & Co., Inc., West Point, PA 19486, USA
19. Human Genetics Branch, National Institute of Mental Health, Bethesda, MD 20892, USA
20. Molecular Genetics Section, Laboratory of Neurogenetics, National Institute on Aging, Bethesda, MD 20892, USA
21. Department of Neurology, University of Michigan, 1500 E Medical Center Dr, Ann Arbor, MI 48109, USA
22. Motor Neuron Disorders Unit, Laboratory of Neurogenetics, National Institute of Neurological Disorders and Stroke, Bethesda, MD 20892, USA
23. Department of Pathology, Penn State College of Medicine, Hershey, PA 17033, USA
24. Department of Neurology, University of Utah School of Medicine, 175 North Medical Drive East, Salt Lake City, UT 84132, USA
25. Department of Neurology, Emory University School of Medicine, Atlanta, GA 30322, USA
26. Department of Molecular Neuroscience and Reta Lila Weston Laboratories, Institute of Neurology, University College London, London, WC1N 3BG, UK
27. Department of Neurology, Columbia University, New York, NY 10032, USA
28. Department of Neurology, Drexel University College of Medicine, Philadelphia, PA 19102, USA
29. Department of Neurology, Temple University, 7602 Central Ave, Philadelphia, PA 19111, USA
30. Department of Neurology, University of Helsinki, Helsinki, FIN-02900, Finland
31. Department of Neurology, Veterans Affairs Boston Healthcare System, Boston, MA 02130, USA
32. Cell Biology and Gene Expression Section, Laboratory of Neurogenetics, National Institute on Aging, Bethesda, MD 20892, USA
33. Department of Geriatrics, Neurosciences and Orthopedics, Center for Geriatric Medicine, Catholic University of Sacred Heart, Rome, 00168, Italy
34. INM, INSERM, University of Montpellier, CHU Nîmes, Nîmes, France
35. Neuromuscular Division and ALS Center, Beth Israel Medical Center, Albert Einstein College of Medicine, New York, NY 01605, USA
36. Department of Neurology, Johns Hopkins University, Baltimore, MD 21287, USA
37. ALS Center, ICS Maugeri, IRCCS, Via Camaldoli, 64, Milan, 20138, Italy
38. Department of Pathology, Haartman Institute/HUSLAB, University of Helsinki and Folkhalsan Research Center (LM), Helsinki, FIN-02900, Finland
39. Data Tecnica International, Glen Echo, MD 20812, USA
40. Department of Clinical Neuroscience, Institute of Neurology, University College London, London, NW2 2PG, UK
41. Discipline of Pathology, Brain and Mind Centre, University of Sydney, Camperdown, NSW 2050, Australia
42. Faculty of Human and Medical Sciences, University of Manchester, Manchester, M13 9PT, UK
43. Department of Neurology, Cleveland Clinic, Cleveland, OH 44195, USA
44. Clinical and Neuropathology Core, Johns Hopkins University, Baltimore, MD 21287, USA
45. Department of Neuroscience, Experimental Neurology and Leuven Research Institute for Neuroscience and Disease, University of California San Diego, 9500 Gilman Drive, La Jolla, CA 92093, USA

© 2021 Johnson JO et al. *JAMA Neurology*.

46. Nash Family Department of Neuroscience, Ronald M. Loeb Center for Alzheimer's Disease, Icahn School of Medicine at Mount Sinai, New York, NY 10029, USA
47. Department of Neurosciences, Experimental Neurology and Leuven Research Institute for Neuroscience and Disease, University of Leuven, Leuven, 3000, Belgium
48. Division of Neurology, Tanz Centre for Research of Neurodegenerative Diseases and Toronto Western Hospital, University of Toronto, Toronto, M5S 3H2, Canada
49. Department of Neurology, Institute for Clinical Neurobiology, University of Würzburg, Würzburg, D-97078, Germany
50. Department of Neurology, Penn State College of Medicine, Hershey, PA 17033, USA
51. VIB, Center for Brain & Disease Research, Laboratory of Neurobiology, University of Leuven, Leuven, 3000, Belgium
52. Department of Pathology and Laboratory Medicine, University of Pennsylvania, Philadelphia, PA 19104, USA
53. Division of Neurology, Sunnybrook Health Sciences Centre, University of Toronto, Toronto, M4N 3M5, Canada
54. Department of Neurology, University of Massachusetts Medical School, Worcester, MA 01605, USA
55. 'Rita Levi Montalcini' Department of Neuroscience, University of Turin, Via Verdi 8, Turin, 10124, Italy
56. Neuroscience Institute of Torino, University of Turin, Turin, 10124, Italy
57. National Institute of Neurological Disorders and Stroke, Bethesda, MD 20892, USA

## The members of the ITALSGEN Consortium are:

Stefania M. Angelocola, MD[1], Francesco P. Ausiello, MD[2], Marco Barberis, BSc[3], Ilaria Bartolomei, MD[4], Stefania Battistini, MD, PhD[5], Enrica Bersano, MD[6], Giulia Bisogni, MD[7], Giuseppe Borghero, MD[8], Maura Brunetti, BSc[9], Corrado Cabona, MD[10], Andrea Calvo, MD, PhD[9,11], Fabrizio Canale, MD[12], Antonio Canosa, MD, PhD[9,11], Teresa A. Cantisani, MD[13], Margherita Capasso, MD[14], Claudia Caponnetto, MD[10], Patrizio Cardinali, MD[1], Paola Carrera, PhD[15], Federico Casale, Bio[9], Adriano Chiò, MD[9,11], Tiziana Colletti, MD[16], Francesca L. Conforti, MD[17], Amelia Conte, MD[7], Elisa Conti, MD[18,19], Massimo Corbo, MD[20], Stefania Cuccu, PsyD[8], Eleonora Dalla Bella, MD[6], Eustachio D'Errico, MD[21], Giovanni DeMarco, PhD[9], Raffaele Dubbioso, MD[2], Carlo Ferrarese, MD[18,19], Pilar M. Ferraro, PhD[10], Massimo Filippi, MD, PhD[22,23,24,25], Nicola Fini, MD[26], Gianluca Floris, MD[8], Giuseppe Fuda, BSc[9], Salvatore Gallone, MD[9], Giulia Gianferrari, MD[26], Fabio Giannini, MD, PhD[5], Maurizio Grassano, MD[9], Lucia Greco, PsyD[27], Barbara Iazzolino, PsyD[9], Alessandro Introna, MD[21], Vincenzo La Bella, MD, PhD[16], Serena Lattante, PhD[28,29], Giuseppe Lauria, MD[6,30], Rocco Liguori, MD, PhD[31], Giancarlo Logroscino, MD, PhD[32,33], Francesco O. Logullo, MD[34], Christian Lunetta, MD[27], Paola Mandich, MD[10,35], Jessica Mandrioli, MD[26], Umberto Manera, MD[9], Fiore Manganelli, MD[2], Giuseppe Marangi, MD[28,29], Kalliopi Marinou, MD[36], Maria Giovanna Marrosu, MD[37], Ilaria Martinelli, MD[26], Sonia Messina, MD, PhD[38], Cristina Moglia, MD, PhD[9,11], Gabriele Mora, MD[36], Lorena Mosca, PhD[39], Maria R. Murru, MD[8], Paola Origone, PhD[10], Carla Passaniti, MD[40], Cristina Petrelli, MD[34], Antonio Petrucci, MD[41], Susanna Pozzi, PsyD[27], Maura Pugliatti, MD, PhD[42], Angelo Quattrini, MD, PhD[43], Claudia Ricci, MD[5], Giulia Riolo, PhD[5], Nilo Riva, MD, PhD[43], Massimo Russo, MD[44], Mario Sabatelli, MD[45], Paolina Salamone, PhD[9], Marco Salivetto, PhD[27], Fabrizio Salvi, MD[4], Marialuisa Santarelli, MD[46], Luca Sbaiz, BSc[3], Riccardo Sideri, MD[36], Isabella Simone, MD[21], Cecilia Simonini, MD[26], Rossella Spataro, MD[16], Raffaella Tanel, MD[47], Gioacchino Tedeschi, MD[40], Anna Ticca, MD[48], Antonella Torriello, MD[49], Stefania Tranquilli, MD[8], Lucio Tremolizzo, MD[18,19], Francesca Trojsi, MD[40], Rosario Vasta, MD[9], Veria Vacchiano, MD[4], Giuseppe Vita, MD, PhD[44], Paolo Volanti, MD[50], Marcella Zollino, MD[51,52], Elisabetta Zucchi, MD[26]

1. Neurology Unit, AV4 ASUR Marche, Fermo, Italy.
2. Department of Neurosciences, Reproductive Sciences and Odontostomatology, University of Naples Federico II, Napoli, Italy.
3. Department of Medical Genetic, Azienda Ospedaliero Universitaria Città della Salute e della Scienza, Torino, Italy.
4. Center for Diagnosis and Cure of Rare Diseases, Department of Neurology, IRCCS Institute of Neurological Sciences, Bologna, Italy.

© 2021 Johnson JO et al. *JAMA Neurology*.

5. Department of Medical, Surgical and Neurological Sciences, University of Siena, Siena, Italy.
6. 3rd Neurology Unit and Motor Neuron Diseases Centre, IRCCS Foundation "Carlo Besta" Neurological Institute, Milano, Italy.
7. NeuroMuscular Omnicentre (NEMO), Serena Onlus, Foundation- Pol. A. Gemelli, Roma, Italy.
8. Neurologic Unit, Monserrato University Hospital, Cagliari University, Cagliari, Italy.
9. `Rita Levi Montalcini` Department of Neuroscience, Amyotrophic Lateral Sclerosis Center, University of Torino, Torino, Italy.
10. Department of Neurosciences, Rehabilitation, Ophthalmology, Genetics and Maternal-Child Sciences, IRCCS Ospedale Policlinico San Martino, Genoa, Italy.
11. Division of Neurology, Azienda Ospedaliero-Universitaria Città della Salute e della Scienza di Torino, Torino, Italy.
12. Department of Advanced Medical and Surgical Sciences, University of Campania "Luigi Vanvitelli", Caserta, Italy.
13. Struttura complessa di Neurofisiopatologia, Azienda Ospedaliera di Perugia, Perugia, Italy.
14. Unit of Neurology, Ospedale Clinicizzato SS Annunziata, Chieti, Italy.
15. Unit of Genomics for the diagnosis of human pathologies, IRCCS San Raffaele Scientific Institute, Milano, Italy.
16. ALS Clinical Research Center, Bi.N.D., University of Palermo, Palermo, Italy.
17. Department of Pharmacy, Health and Nutritional Sciences, University of Calabria, Rende, Italy.
18. Neurology Unit, "San Gerardo" hospital, Monza, Italy.
19. School of Medicine and Surgery and Milan Center for Neuroscience (NeuroMI), University of Milano-Bicocca, Milano, Italy.
20. Department of Neurorehabilitation Sciences, Casa Cura Policlinico, Milano, Italy.
21. Department of Basic Medical Sciences, Neurosciences and Sense Organs, University of Bari “Aldo Moro”, Policlinic, Bari, Italy.
22. Neurology Unit and Rehabilitation Unit, IRCCS ”San Raffaele Scientific Institute”, Milano, Italy.
23. Neuroimaging Research Unit, Division of Neuroscience, IRCCS ”San Raffaele Scientific Institute”, Milano, Italy.
24. Neurophysiology Service, IRCCS ”San Raffaele Scientific Institute”, Milano, Italy.
25. Vita-Salute San Raffaele University, Milano, Italy.
26. Department of Neurosciences, Ospedale Civile S. Agostino Estense, Azienda Ospedaliero Universitaria di Modena, Modena, Italy.
27. NEMO Clinical Center Milano, Fondazione Serena Onlus, Milano, Italy.
28. Genetica Medica, Fondazione Policlinico Universitario A. Gemelli IRCCS, Roma, Italy.
29. Dipartimento Universitario Scienze della Vita e Sanità Pubblica, Sezione di Medicina Genomica, Università Cattolica del Sacro Cuore Facoltà di Medicina e Chirurgia, Roma, Italy.
30. Department of Biomedical and Clinical Sciences Luigi Sacco, University of Milan, Milano, Italy.
31. Department of Biomedical and Neuromotor Sciences, University of Bologna, Bologna, Italy.
32. Department of Basic Medical Sciences, Neuroscience and Sense Organs, University of Bari Aldo Moro, Bari, Italy.
33. Center for Neurodegenerative Diseases and the Aging Brain, Department of Clinical Research in Neurology, Pia Fondazione Cardinale G Panico, Tricase, Italy.
34. Neurology Unit, AV3, ASUR Marche, Macerata, Italy.
35. Medical Genetics Unit, IRCCS Ospedale Policlinico San Martino, Genoa, Italy.
36. Department of Neurorehabilitation, Istituti Clinici Scientifici Maugeri IRCCS, Institute of Milan, Milano, Italy.
37. Department of Neurology, Azienda Universitario Ospedaliera di Cagliari and University of Cagliari, Cagliari, Italy.
38. NEuroMuscular Omnicentre (NEMO) Sud, Fondazione Aurora, OUC Neurology and Neuromuscular Disorders, University of Messina, Italy, Messina, Italy.
39. Department of Laboratory Medicine, Medical Genetics, Niguarda Ca' Granda Hospital, Milano, Italy.
40. Department of Advanced Medical and Surgical Sciences, University of Campania "Luigi Vanvitelli", Napoli, Italy.
41. Neurology Department, San Camillo Hospital, Roma, Italy.
42. Department of Biomedical and Surgical Sciences, Section of Neurological, Psychiatric and Psychological Sciences, University of Ferrara, Ferrara, Italy.
43. Department of Neurology, IRCCS ”San Raffaele Scientific Institute”, Milano, Italy.
44. OUC Neurology and Neuromuscular Disorders, University of Messina, Messina, Italy.
45. NeuroMuscular Omnicentre (NEMO) - Fondazione Policlinico Universitario A. Gemelli, Università Cattolica del Sacro Cuore, Roma, Italy.
46. Department of Medicine, Azienda Complesso Ospedaliero, San Filippo Neri, Roma, Italy.
47. Operative Unit of Neurology, S. Chiara Hospital, Trento, Italy.
48. Department of Neurology, Azienda Ospedaliera San Francesco, Nuoro, Italy.
49. ALS Center, Operative Unit of Neurology, AOU “San Giovanni di Dio e Ruggi d’Aragona”, Salerno, Italy.
50. Neurorehabilitation Unit – ALS Center, Istituti Clinici Scientifici (ICS) Maugeri, Mistretta, Italy.

© 2021 Johnson JO et al. *JAMA Neurology*.

51. Sezione di Medicina Genomica, Dipartimento Scienze della Vita e Sanità Pubblica, Facoltà di Medicina e Chirurgia, Università Cattolica Sacro Cuore, Roma, Italy.
52. Unità di Genetica Medica, Fondazione Policlinico Universitario A. Gemelli IRCCS, Roma, Italy.

© 2021 Johnson JO et al. *JAMA Neurology*.

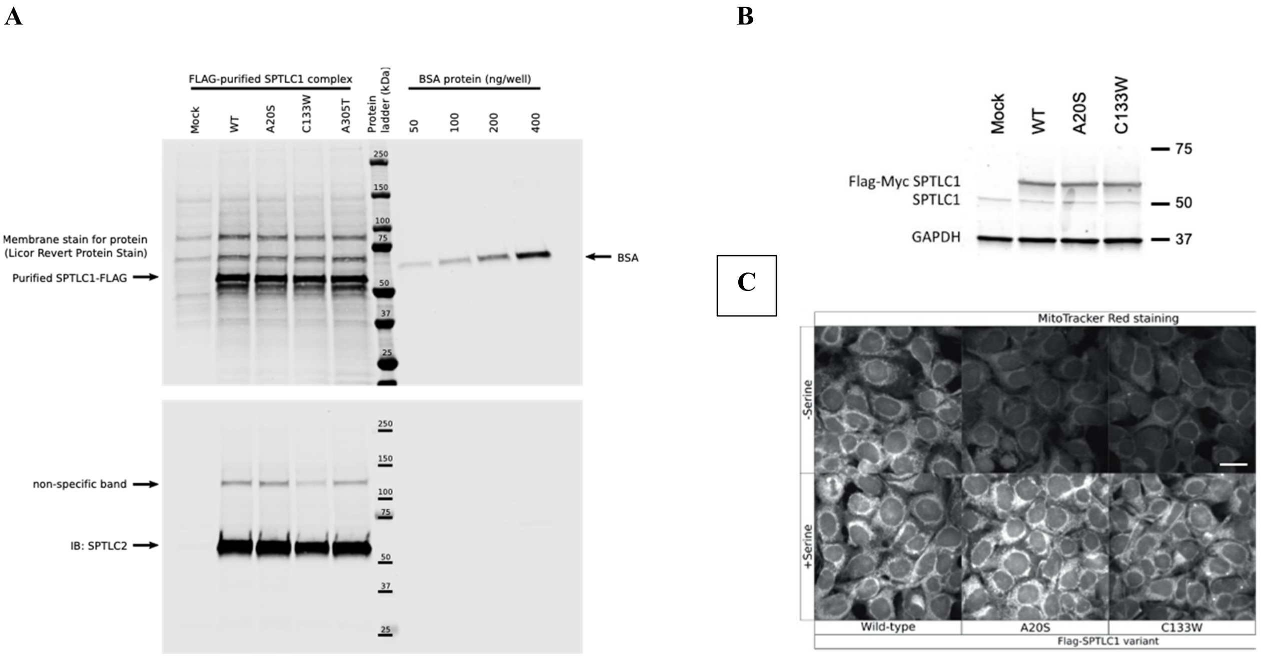


**eFigure 1.** Assays of SPT activity and mitochondrial cellular phenotypes in cell models

**(A)** Western blot of purified SPTLC1-FLAG proteins showing that the purified complex contains SPTLC2**. (B)** Western blot showing comparable SPTLC1 expression across all lines of HEK293 cells stably overexpressing wild-type (WT) or mutant proteins. Exogenous SPTLC1 expression was qualitatively estimated to be ~10x higher than endogenous levels. **(C)** Representative image from CX7 LZR high-content analysis instrument of MitoTracker staining in wild-type and mutant SPTLC1 cells with and without treatment with 400 μM L-serine for 48 hours. Treatment with 400 μM L-serine rescued mitochondrial abnormalities in cells overexpressing mutant SPTLC1. Scale bar = 25μm.

© 2021 Johnson JO et al. *JAMA Neurology*.

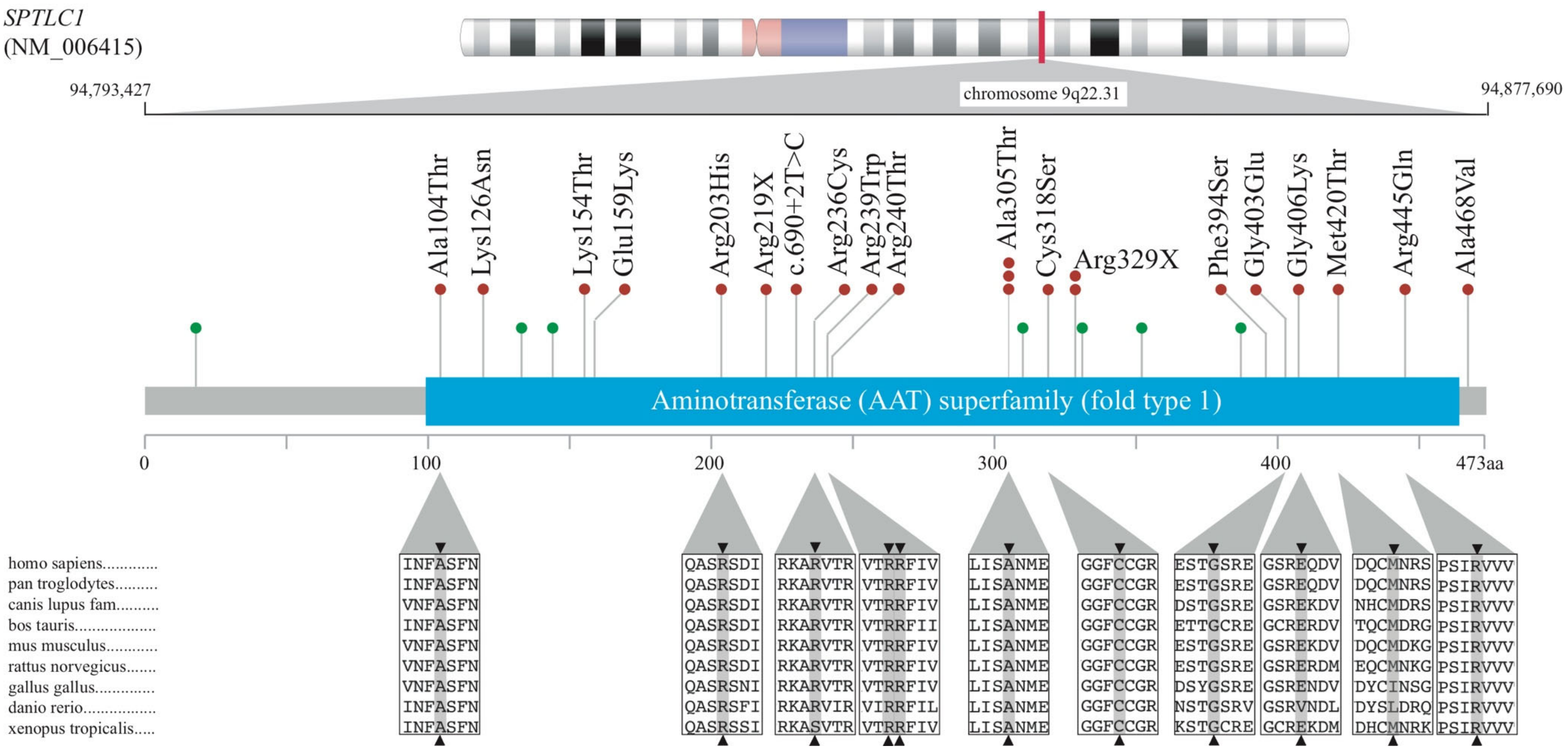


**eFigure 2.** *SPTLC1* mutations detected in adult-onset ALS patients

The top panel is a graphical representation showing the domains of SPTLC1 based on ENST00000262554.2. Mutations detected in ALS cases are indicated in red, and mutations previously described to cause HSAN1 are shown in green. The p.Ala305Thr variant was found in three ALS patients, and the other variants were found in single cases of adult-onset ALS. The middle panel shows the conservation of amino acid residue across species generated using the Clustal Omega online tool.

© 2021 Johnson JO et al. *JAMA Neurology*.

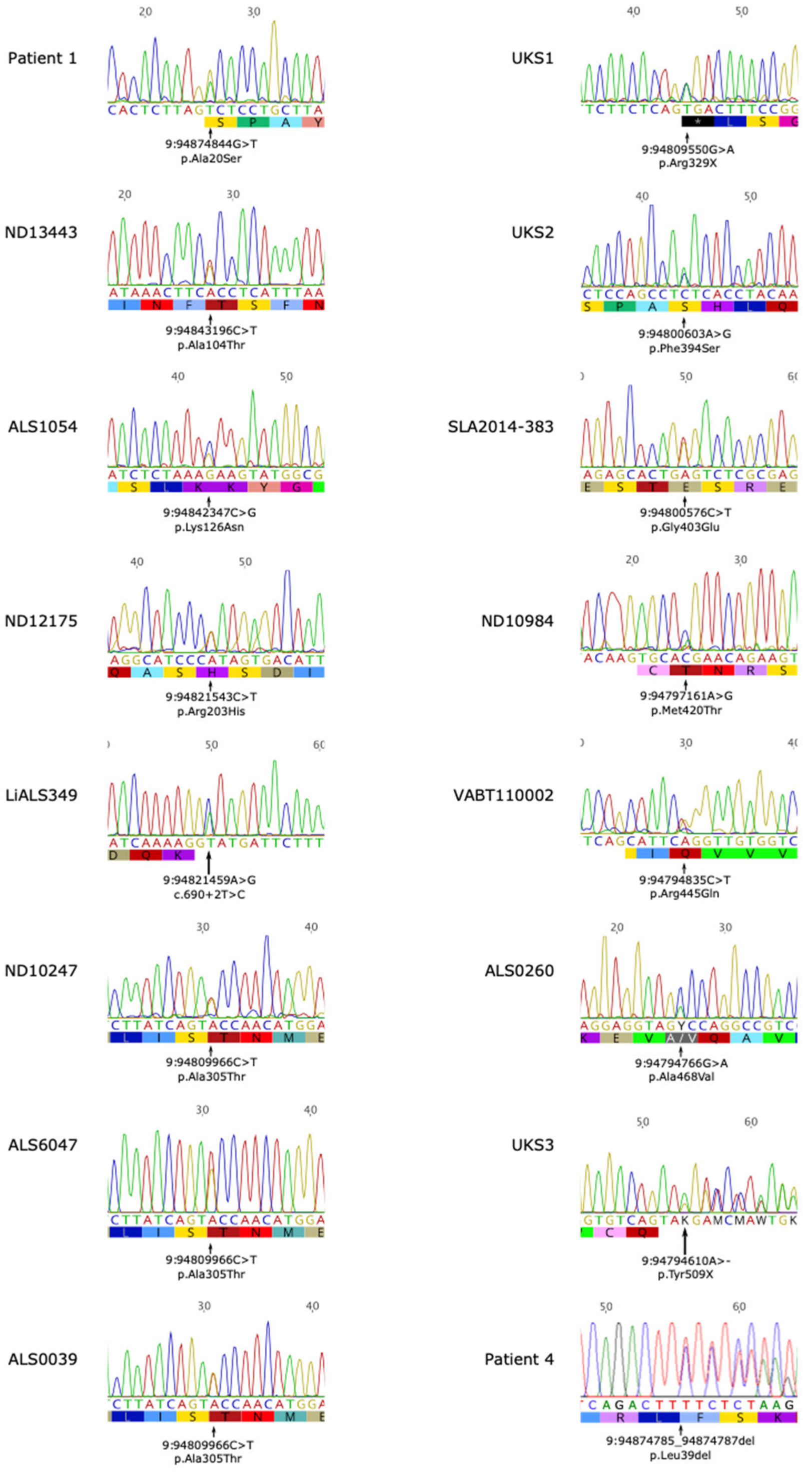


**eFigure 3.** Chromatograms of *SPTLC1* variants identified in patients

© 2021 Johnson JO et al. *JAMA Neurology*.

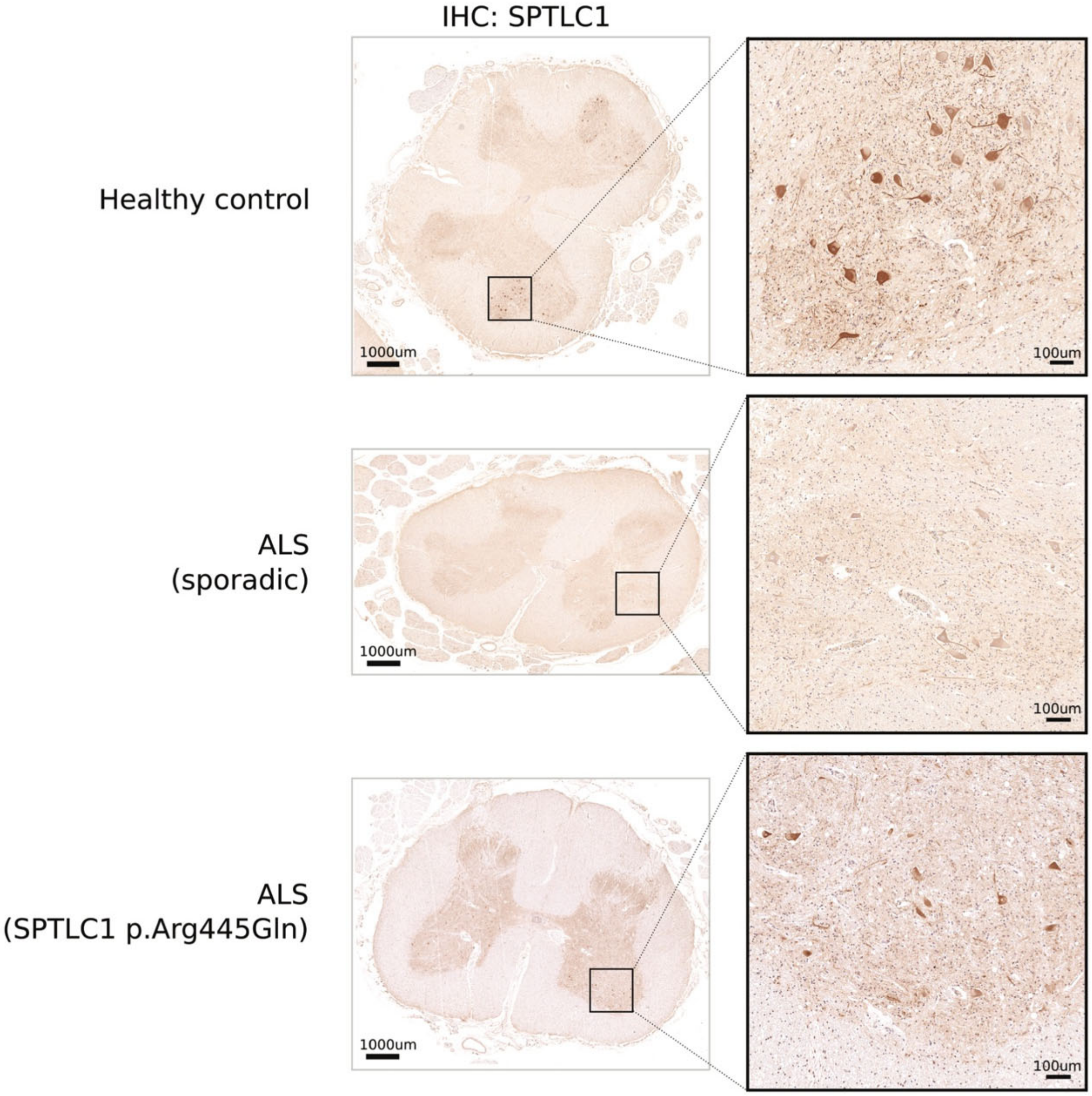


**eFigure 4.** Immunohistochemistry of SPTLC1 in the lumbar spinal cord

The control spinal cord (top panel) exhibits SPLTC1 immunoreactivity in the lamina IX motor neurons of the anterior horns. The spinal cord from a subject with sporadic ALS (middle panel) shows weaker immunoreactivity in the same region, consistent with a decrease in the number and size of surviving motor neurons. The spinal cord from an ALS case carrying the p.Arg445Gln mutation in SPTLC1 (bottom panel) shows a similar diminished staining pattern as the sporadic ALS sample. Scale bars in magnified inset represent 100 μm.

© 2021 Johnson JO et al. *JAMA Neurology*.

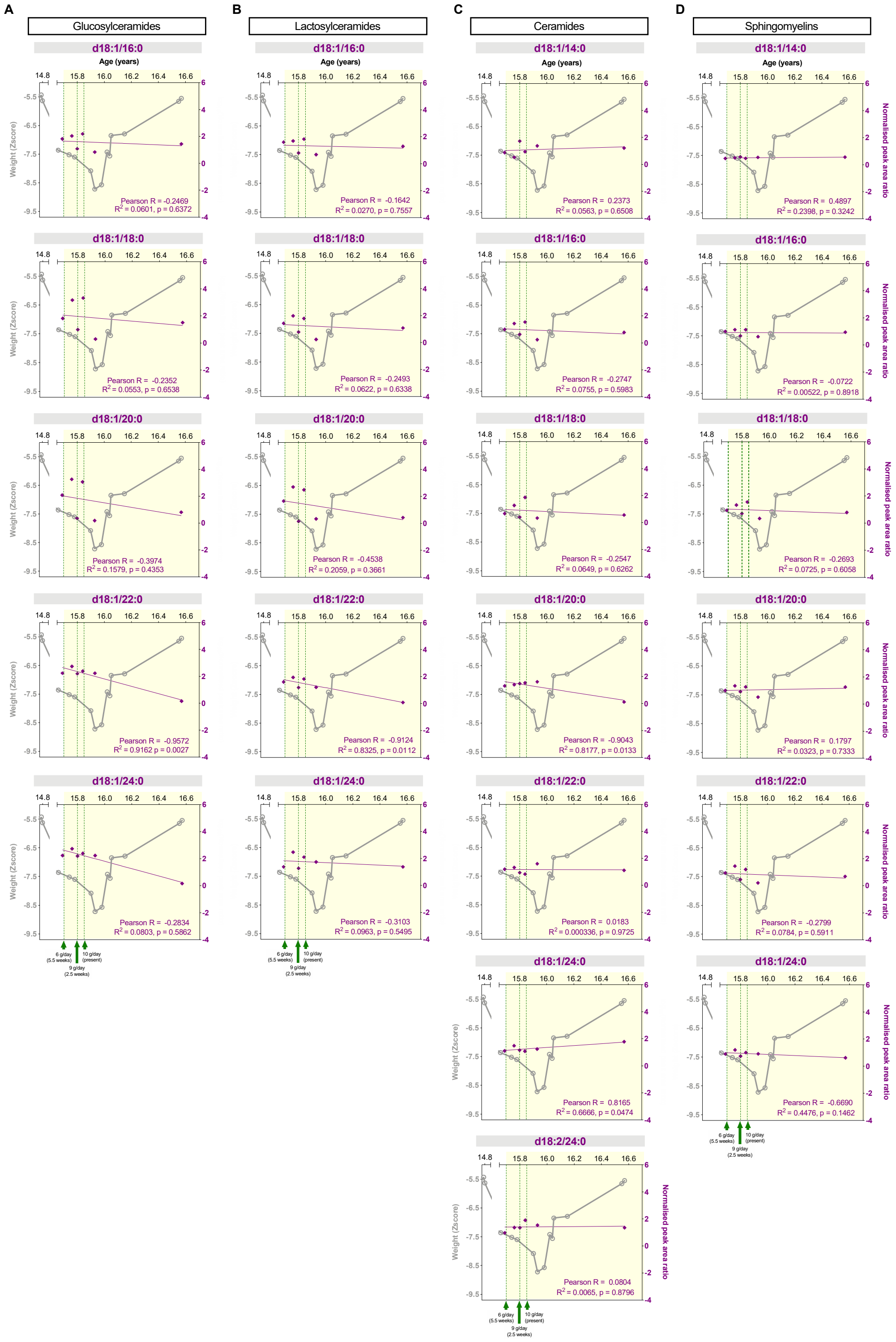


**eFigure 5.** Time-series changes of complex sphingolipids in patient 2 on high-dose oral serine supplementation

Glucosylceramides, lactosylceramides, ceramides, and sphingomyelins levels were measured in patient 2 before and throughout serine supplementation. The purple lines represent the linear regression lines over time, with the 95% confidence interval demarcated by dotted lines. Pearson R = regression slope, $R^2$ = slope correlation and p = significance of correlation. Green arrows show the serine doses, and the duration of each dosage is given in brackets. The x-axis shows the age in years, the left y-axis shows the z-score weights, and the right y-axis is the normalized peak area ratio from lipid mass spectra on a $\log_{10}$ scale.

© 2021 Johnson JO et al. *JAMA Neurology*.

**eTable 1.** Demographic and clinical features of adult-onset ALS cases screened for *SPTLC1* mutations

| | | ALS cases (n = 5607)* | | Control subjects (n=5,710) |
|---|---|---|---|---|
| Mean age at onset/sampling (SD) | | 57.4 (14.9) | | 86.1 (6.9) |
| Female (%) | | 2,017 (42.7%) | | 3,370 (59.0%) |
| Site of onset: | | | | |
| Bulbar (%) | | 979 (26.7%) | | NA |
| Spinal (%) | | 2,693 (73.3%) | | NA |
| Familial disease (%) | | 2,322 (46.1%) | | NA |

ALS cases include samples from the USA (n = 1,922), Italy (n = 1,295), Finland (n = 306), Germany (n = 243), Turkey (n = 651), United Kingdom (n = 851) and other countries (n = 128); Control samples include samples from the USA (n = 4,647), Italy (n = 763) and Finland (n = 300); Control participants consisted of samples that had undergone whole-genome sequencing (n = 300 Finnish and n = 677 Italian) and samples that had undergone exome sequencing as part of the Alzheimer's Disease Sequencing Project (n = 4,647 Americans). Clinical data was missing for age at onset (n = 1,707), sex (n = 882), site of onset (n = 1,935), family status (n = 567) and country (n = 704). *Turkish ALS cases (n = 621) are not included.

© 2021 Johnson JO et al. *JAMA Neurology*.

**eTable 2.** Primer sequences used for Sanger sequencing and mutagenesis of *SPTLC1*

PCR and sequencing primers

| | |
|---|---|
| 1 forward | GGTCTCAGGCCACAAATCC |
| 1 reverse | CACCTGACCCCGTCCAG |
| 2 forward | GAGCCACCACAAATCCTTTC |
| 2 reverse | AAATTTTATGTGCAGGTGTTAGAAG |
| 3 forward | CTCCTGGGAACTTCAAAATG |
| 3 reverse | GAGTGACAGCGAACTTGAGAATAG |
| 4 forward | CCCAGCCTGCCTTTGTC |
| 4 reverse | TTGTTGTGCCTACCACCAAG |
| 5 forward | GCCCAGCCTAAAATTGAATC |
| 5 reverse | TGGGTTAAAGAATAGGAAATGTTTG |
| 6 forward | TGAAACCTTTGGTGAGGTG |
| 6 reverse | TGGCCATAAGGTGAAAGTATTG |
| 7 forward | AGACTTGGCTCTCCAACACC |
| 7 reverse | CATGGATGAAGAGCAAACAATG |
| 8 forward | CGGTCATAAACAAAAGGCAG |
| 8 reverse | AAGTGCTGGGGAATATGTAACAG |
| 9 forward | GGCCTAGCAGAATGGAACTAC |
| 9 reverse | GAATTCACAAGTATTGAGAGTCAGC |
| 10 forward | GAGGTGACCAATGGAGAAATTAG |
| 10 reverse | TCTCTAGGCTTGAAGTTGGC |
| 11 forward | TGGCAAATAATTTCTCCAGTAAG |
| 11 reverse | TTGTTCTAGAGAACTGGTGCC |
| 12 forward | CTGGTCAAACTGACCCAAGC |
| 12 reverse | AAATTGATGAACTTGATCTGGG |
| 13 forward | AAATTGCTTGGGGCAAGG |
| 13 reverse | GAGCGATCTTCAAGCCTGG |
| 14 forward | TTCAAAAGATAACGTATTTCATAGAGC |
| 14 reverse | GTTGCAGGATTTCAGCGTTC |
| 15 forward | TGAAGCTCCAGTTTTAAACGAC |
| 15 reverse | TGACCTCGTGATCCACCTG |

© 2021 Johnson JO et al. *JAMA Neurology*.

**eTable 3.** *SPTLC1* mutations identified in adult-onset ALS cases

| SNP information | | | No. cases | | Population frequency | | Prediction and conservation algorithms | | |
|---|---|---|---|---|---|---|---|---|---|
| | | | | | gnomAD | | Prediction | Conservation | dbscSNV |
| 9:94843196C>T | p.Ala104Thr | | 1 | | $1{\cdot}1\times10^{-5}$ | | damaging | conserved | NA |
| 9:94842347C>G | p.Lys126Asn | | 1 | | 0 | | damaging | not conserved | NA |
| 9:94830347T>G | p.Lys154Thr | | 1 | | 0 | | damaging | not conserved | NA |
| 9:94830333C>T | p.Glu159Lys | | 1 | | 0 | | damaging | conserved | NA |
| 9:94821543C>T | p.Arg203His | | 1 | | $1{\cdot}1\times10^{-5}$ | | damaging | conserved | NA |
| 9:94821496G>A | p.Arg219X | | 1 | | $2{\cdot}2\times10^{-5}$ | | damaging | conserved | NA |
| 9:94821459A>G | c.690+2T>C | | 1 | | 0 | | NA | conserved | 0·994 |
| 9:94817761G>A | p.Arg236Cys | | 1 | | 0 | | damaging | not conserved | NA |
| 9:94817752G>A | p.Arg239Trp | | 1 | | 0 | | damaging | not conserved | NA |
| 9:94817749G>A | p.Arg240Cys | | 1 | | $1{\cdot}1\times10^{-5}$ | | damaging | conserved | NA |
| 9:94809966C>T | p.Ala305Thr | | 3 | | $1{\cdot}1\times10^{-5}$ | | damaging | conserved | NA |
| 9:94809927A>T | p.Cys318Ser | | 1 | | $1{\cdot}1\times10^{-5}$ | | damaging | conserved | NA |
| 9:94809550G>A | p.Arg329X | | 2 | | $1{\cdot}1\times10^{-5}$ | | damaging | conserved | 0·15 |
| 9:94800603A>G | p.Phe394Ser | | 1 | | 0 | | damaging | conserved | NA |
| 9:94800576C>T | p.Gly403Glu | | 1 | | 0 | | damaging | conserved | NA |
| 9:94800568C>T | p.Glu406Lys | | 1 | | 0 | | damaging | conserved | NA |
| 9:94797161A>G | p.Met420Thr | | 1 | | 0 | | damaging | conserved | NA |
| 9:94794835C>T | p.Arg445Gln | | 1 | | $2{\cdot}2\times10^{-5}$ | | damaging | conserved | NA |
| 9:94794766G>A | p.Ala468Val | | 1 | | $1{\cdot}1\times10^{-5}$ | | damaging | conserved | NA |
| 9:94794610Adel | p.Tyr509X | | 1 | | 0 | | damaging | conserved | NA |

Variant position is in build 19; amino acid change is based on the canonical transcript NP_006406, except for p.Tyr509X which is based on NP_001268232; No. cases, number of ALS cases carrying the variant; only variants with frequency less than $3{\cdot}3\times10^{-5}$ are shown; gnomAD, frequency shown is the maximum in either the Finnish or the non-Finnish European populations (non-neurological samples, version 2·1); 'damaging' means the variant is designated as such by at least four out of five prediction algorithms consisting of FATHMM, M-CAP, MetaLR, MetaSVM and VEST3; 'conserved' means the position is considered as such by the GERP++, phastCons100way_vertebrate, phyloP100way_vertebrate, and SiPhy_29way algorithms; dbscSNV adaptive boosting score greater than 0·6 indicates a deleterious splice mutation; NA, not available.

© 2021 Johnson JO et al. *JAMA Neurology*.

**eTable 4.** Demographic and clinical features of adult-onset ALS cases carrying *SPTLC1* mutations

| Sample and mutation | | | | | Clinical and demographic features | | | | |
|---|---|---|---|---|---|---|---|---|---|
| *Sample* | *Mutation* | | *Sex* | *Age* | *Onset* | *Type* | *C9orf72* | *Sensory/ autonomic* | *Country* |
| ND13443 | p.Ala104Thr | | M | 55 | spinal | sporadic | no | none | USA |
| ALS1054 | p.Lys126Asn | | F | 66 | spinal | familial | no | none | Italy |
| ALS855 | p.Lys154Thr | | M | 31 | spinal | sporadic | no | none | Turkey |
| ALS590 | p.Glu159Lys | | M | 34 | spinal | sporadic | no | none | Turkey |
| ND12175 | p.Arg203His | | M | 38 | spinal | sporadic | no | none | USA |
| A14LIALS68 | p.Arg219X | | M | 49 | spinal | sporadic | no | none | Finland |
| A14LIALS349 | c.690+2T>C | | M | 57 | spinal | familial | yes | none | Finland |
| B555 | p.Arg236Cys | | M | 51 | spinal | sporadic | no | NA | Italy |
| ALS1569 | p.Arg239Trp | | M | 41 | spinal | sporadic | no | none | Turkey |
| SLA2010-83 | p.Arg240Cys | | M | 73 | bulbar | sporadic | no | none | Italy |
| ND10247 | p.Ala305Thr | | M | 76 | bulbar | familial | no | none | USA |
| AUS145-010335 | p.Ala305Thr | | F | 58 | NA | familial | no | NA | Australia |
| ALS0039 | p.Ala305Thr | | M | 64 | spinal | familial | no | none | UK |
| SLA2011-105 | p.Cys318Ser | | M | 58 | spinal | sporadic | no | none | Italy |
| UKS1 | p.Arg329X | | M | 62 | spinal | sporadic | no | none | UK |
| ALS668 | p.Arg329X | | F | 56 | spinal | sporadic | no | NA | Turkey |
| UKS2 | p.Phe394Ser | | M | 54 | spinal | sporadic | no | none | UK |
| SLA2014-383 | p.Gly403Glu | | F | 75 | bulbar | sporadic | no | none | Italy |
| A356 | p.Glu406Lys | | F | 52 | spinal | sporadic | no | NA | Italy |
| ND10984 | p.Met420Thr | | M | 52 | spinal | sporadic | no | none | USA |
| VABT110002 | p.Arg445Gln | | M | 72 | spinal | sporadic | no | none | USA |
| ALS0260 | p.Ala468Val | | M | 81 | spinal | sporadic | no | none | Israel |
| UKS3 | p.Tyr509X | | F | 56 | general | sporadic | no | none | UK |

Mutation description is based on the canonical NM_006415 transcript, except for p.Tyr509X which is based on NM_001281303·1; M refers to male and F to female; age refers to age at onset; onset refers to site where symptoms manifested initially; general means generalized onset; *C9orf72* refers to the pathogenic repeat expansion in that gene; country refers to population of origin of the sample. All patients carrying an *SPTLC1* mutation were of European ancestry; NA means not available; samples starting with ND are available from the NINDS Repository at the Coriell Institute for Medical Research.

© 2021 Johnson JO et al. *JAMA Neurology*.

**eTable 5.** Complex sphingolipid plasma levels in ALS cases and controls

Values represent normalized peak area ratios. In patient 2, the measurements were made before serine supplementation and 10.5 months after commencing serine.

| **Sample** | **Glucosylceramides** | | | | | | **Lactosylceramides** | | | | |
|---|---|---|---|---|---|---|---|---|---|---|---|
| | **d18:1/ 16:0** | **d18:1/ 18:0** | **d18:1/ 20:0** | **d18:1/ 22:0** | **d18:1/ 24:0** | | **d18:1/ 16:0** | **d18:1/ 18:0** | **d18:1/ 20:0** | **d18:1/ 22:0** | **d18:1/ 24:0** |
| Patient 1 | 0.92 | 1.24 | 0.78 | 0.90 | 1.07 | | 0.83 | 0.62 | 0.68 | 0.60 | 1.08 |
| Patient 1_Mother | 0.51 | 0.29 | 0.08 | 0.49 | 0.53 | | 0.60 | 0.60 | 0.16 | 0.32 | 0.72 |
| Patient 1_Brother | 0.50 | 0.40 | 0.36 | 1.27 | 1.35 | | 0.65 | 0.59 | 0.34 | 0.81 | 1.72 |
| | | | | | | | | | | | |
| Patient 2 (before serine) | 1.82 | 1.82 | 2.08 | 2.24 | 1.81 | | 1.60 | 1.43 | 1.65 | 1.60 | 1.38 |
| Patient 2 (after serine) | 1.44 | 1.51 | 0.81 | 0.16 | 1.94 | | 1.29 | 1.08 | 0.43 | 0.08 | 1.38 |
| | | | | | | | | | | | |
| Patient 3 | 1.03 | 0.61 | 0.34 | 1.40 | 1.24 | | 0.61 | 0.45 | 0.40 | 1.44 | 1.16 |
| Patient 3_Mother | 0.55 | 0.24 | 0.14 | 0.60 | 0.96 | | 0.45 | 0.38 | 0.15 | 0.40 | 0.58 |
| Patient 3_Father | 0.60 | 0.29 | 0.05 | 0.55 | 0.56 | | 0.97 | 0.55 | 0.23 | 0.48 | 0.56 |
| | | | | | | | | | | | |
| Control Male 1 (> 50 yrs) | 1.25 | 1.31 | 1.57 | 1.55 | 1.13 | | 1.40 | 1.73 | 1.55 | 1.68 | 0.65 |
| Control Male 2 (> 50 yrs) | 0.44 | 0.23 | 0.21 | 0.83 | 0.71 | | 0.59 | 0.35 | 0.36 | 0.91 | 0.89 |
| Control Female (16 yrs) | 1.02 | 0.98 | 1.65 | 1.35 | 1.28 | | 0.96 | 0.83 | 1.51 | 1.05 | 0.89 |
| Control Male (16 yrs) | 0.35 | 0.32 | 0.25 | 1.13 | 1.39 | | 0.43 | 0.18 | 0.20 | 0.47 | 0.78 |

| **Sample** | **Ceramides** | | | | | | | | **Sphingomyelins** | | | | | |
|---|---|---|---|---|---|---|---|---|---|---|---|---|---|---|
| | **d18:1/ 14:0** | **d18:1/ 16:0** | **d18:1/ 18:0** | **d18:1/ 20:0** | **d18:1/ 22:0** | **d18:1/ 24:0** | **d18:2/ 24:0** | | **d18:1/ 14:0** | **d18:1/ 16:0** | **d18:1/ 18:0** | **d18:1/ 20:0** | **d18:1/ 22:0** | **d18:1/ 24:0** |
| Patient 1 | 0.83 | 0.60 | 0.48 | 0.87 | 0.80 | 1.13 | 1.24 | | 0.92 | 0.95 | 0.88 | 1.32 | 0.59 | 0.80 |
| Patient 1_Mother | 0.19 | 0.38 | 0.28 | 0.73 | 0.71 | 0.99 | 0.68 | | 1.43 | 0.96 | 0.98 | 1.18 | 0.41 | 0.90 |
| Patient 1_Brother | 1.25 | 0.35 | 0.25 | 0.58 | 1.17 | 1.65 | 0.77 | | 1.03 | 0.81 | 0.40 | 0.74 | 0.46 | 1.29 |
| | | | | | | | | | | | | | | |
| Patient 2 (before serine) | 0.87 | 1.01 | 0.67 | 1.31 | 1.19 | 1.11 | 0.97 | | 0.46 | 1.00 | 0.95 | 1.01 | 0.95 | 0.90 |
| Patient 2 (after serine) | 1.22 | 0.78 | 0.56 | 0.12 | 1.11 | 1.79 | 1.35 | | 0.56 | 0.94 | 0.79 | 1.26 | 0.69 | 0.62 |
| | | | | | | | | | | | | | | |
| Patient 3 | 0.97 | 0.61 | 0.33 | 1.42 | 1.63 | 1.68 | 2.18 | | 0.53 | 0.67 | 0.54 | 0.92 | 0.53 | 0.93 |
| Patient 3_Mother | 0.66 | 0.44 | 0.28 | 0.67 | 1.07 | 0.97 | 1.13 | | 1.06 | 0.62 | 0.42 | 1.08 | 0.40 | 0.72 |
| Patient 3_Father | 0.99 | 0.26 | 0.27 | 0.70 | 1.18 | 0.71 | 0.55 | | 0.72 | 0.94 | 0.60 | 0.75 | 0.29 | 0.88 |
| | | | | | | | | | | | | | | |
| Control Male 1 (> 50 yrs) | 1.14 | 1.29 | 0.71 | 1.33 | 0.95 | 1.84 | 1.15 | | 0.97 | 1.59 | 1.34 | 1.52 | 1.86 | 2.46 |
| Control Male 2 (> 50 yrs) | 1.20 | 0.46 | 0.32 | 0.92 | 1.62 | 1.10 | 0.94 | | 0.79 | 0.71 | 0.41 | 0.62 | 0.31 | 1.22 |
| Control Female (16 yrs) | 0.44 | 0.66 | 0.59 | 0.80 | 0.99 | 0.93 | 0.89 | | 0.57 | 0.76 | 0.82 | 1.10 | 1.25 | 0.76 |
| Control Male (16 yrs) | 1.41 | 0.32 | 0.35 | 0.83 | 1.09 | 1.10 | 0.89 | | 0.94 | 0.58 | 0.33 | 0.84 | 0.61 | 0.85 |

© 2021 Johnson JO et al. *JAMA Neurology*.

## eReferences.


1. N. Sobreira, F. Schiettecatte, D. Valle, A. Hamosh, GeneMatcher: a matching tool for connecting investigators with an interest in the same gene. *Hum Mutat.* 2015;36:928-930.
2. C. B. Brady, K. T. Trevor, T. D. Stein, E. Y. Deykin, S. D. Perkins, J. G. Averill, L. Guilderson, A. C. Mckee, S. W. Renner, N. W. Kowall, The Department of Veterans Affairs Biorepository Brain Bank: A national resource for amyotrophic lateral sclerosis research. *Amyotroph. Lateral Scler. Front. Degener.* 2013;14:591-597.
3. R. Chia, S. Haddock, A. Beilina, I. N. Rudenko, A. Mamais, A. Kaganovich, Y. Li, R. Kumaran, M. A. Nalls, M. R. Cookson, Phosphorylation of LRRK2 by casein kinase 1alpha regulates trans-Golgi clustering via differential interaction with ARHGEF7. *Nat Commun.* 2014;5:5827.
4. M. F. Rutti, S. Richard, A. Penno, A. von Eckardstein, T. Hornemann, An improved method to determine serine palmitoyltransferase activity. *J Lipid Res.* 2009;50:1237-1244.


© 2021 Johnson JO et al. *JAMA Neurology*.
